# Supplementary material for: Multicentennial cycles in continental demography synchronous with solar activity and climate stability
Source: Nat Commun. 2024 Nov 26;15:10248. doi: 10.1038/s41467-024-54474-w (PMC11599897; doi:10.1038/s41467-024-54474-w)
Supplement: Supplementary file 1 — Supplementary Information [file 41467_2024_54474_MOESM1_ESM.pdf]

# Supplementary materials

## Multicentennial cycles in continental demography synchronous with solar activity and climate stability

Kai Wirtz, Nicolas Antunes, Aleksandr Diachenko, Julian Laabs, Carsten Lemmen, Rowan McLaughlin, Gerrit Lohmann, Eduardo Zorita, Detlef Gronenborn

## S1 Comparison to calibration artifacts

We tested for possible artificial synchronization due to variable slopes in the calibration curve [1, 2] in two ways. First, we generated 5 $10^5$ random artificial population growth (RGR) trajectories, for which in average $N$=12,000 $^{14}$C–dates were distributed following a scheme for emulating a sequence of (growth) booms and busts similar to the observations: $N_p$ boom times were randomly drawn in the interval 9–2 ka $^{14}$C age (to fit our study period after calibration). For each boom, 0.8 $N/N_p$ dates were normally distributed using a standard deviation $\delta T$. $N_p$ and $\delta T$ were drawn from a normal density function using the $R$ expression $x \cdot \mathrm{rnorm}(1, 1, 0.5)$ with $x$=11 for $N_p$ and $x$=350 a for $\delta T$, respectively. The remaining 0.2 $N$ dates were generated following a flat distribution.

The second test is similar to the one proposed by [3]: we calculated RGR for a distribution of 5 $10^4$ $^{14}$C dates with linearly increasing density from 9 to 2 ka $^{14}$C age including calibration and SPD generation, and then subtracted the linear increase rate. Residual RGR values represent the artificial effect of variable slopes of the IntCal20 calibration curve on SPDs.

In both cases, RGR deviations from a baseline do in general not coincide with the (growth) booms and busts inherent to the European growth pattern. The second method produced an artificial RGR trajectory at higher temporal resolution, which displays multicentennial modes at $(200\,\mathrm{a})^{-1}$ and $(360\,\mathrm{a})^{-1}$ also inherent to the solar forcing but much faster than the cycle periods of 500 and 680 a typical for Europe or South America (Fig. 3a,c). While faster centennial cycling is found for other continents such as North America, amplitudes of artificial RGR cycles are much smaller than the reconstructed ones. Only for Europe, a modest bust at 6 ka BP and a boom at 4.8 ka BP may be regarded as potentially enhanced by calibration. The other 13–15 booms/busts in continental RGR cycles much exceed artificial fluctuations

both by amplitude and duration, which is also reflected in negative trim correlations between the artificial and reconstructed trajectories as shown for the continents with highest data density, Europe and North America (Fig. S16). We hence conclude that our results are not critically affected by the non-uniform steepness of the IntCal20 curve.

# S2 Solar influence on climate fluctuations

Fluctuations of incoming solar radiation have been indicated to leave a relatively weak impact on near-surface global mean temperature both in modelling studies and data analyses [4–7]. Compared with a more uniform warming due to increasing greenhouse gases, the solar-induced warming pattern is more centered over the subtropics through an increased flux of shortwave radiation in cloud-free regions. This (sub)tropical warming promotes a latitudinal temperature gradient in the stratosphere and, concomitantly, enhances westerly winds in the lower stratosphere and troposphere. The interaction between ultraviolet radiation and ozone in the stratosphere is connected with a downward propagation of stratospheric events [8, 9], which alters the position and strength of the mid-latitude storm track over Europe [10–12]. Although the influence of solar radiation variations on climate is heterogenous [7, 13], significant impacts were detected on regional scales [10, 14–17]. For Holocene Europe, solar activity has been linked to moisture transport and the frequency of floods [16, 18–20], but with different effect sign likely depending on the location [20, 21]. During periods of low solar activity, the southward shift of storm tracks favors enhanced meridional flow and hydrological events by atmospheric blocking [21–23]. Atmospheric blocking has been linked to weather extremes [23], or reduced ocean surface temperature [22]. The mechanism appears to be linked to the El Niño Southern Oscillation (ENSO) signal [24], via teleconnections and/or parallel solar forcing [25]. For example, phases of lower solar activity display a higher frequency of El Niño events [25], with a higher tendency of short-term weather extremes. However, the effect of ENSO on European climate at an interannual scale changes its sign at the multidecadal-to-centennial scale [26]. Based on this finding, we here hypothesize that lower solar activity may on a short timescale enhance the likelihood of weather extremes but on a long timescale reduce variations in the mean climate state.

# S3 Socio-technological expansions

Many of the identified 'boom' and 'bust' phases of continental (relative) growth can be connected to socio-technological changes. However, these changes did not necessarily affect the whole of Europe at a given time, but were part of more complex spatio-temporal trajectories of European prehistory [27, 28]. A much aggregated overview of socio-cultural phases is presented in Tab. S3.

The two most prominent and researched (relative) growth phases in our time-series mark the onset of the Neolithic in Eastern and Central Europe at 7.9 and 7.4–7.2 ka BP, respectively. These events were part of a profound socio-technological innovation in subsistence efficiency, which includes sedentism, larger group size, and domesticated crop plants and animals. The third prominent growth peak at 4.9 ka BP was simultaneous with an increase of pastoralism and creation of new communication networks. All three

socio-technological changes in the 8th and early 5th millennia BP arrived in Central and Western Europe from the East by migrations and were thus accompanied by genetic admixture. The first migration wave was driven by farmers carrying the entire Neolithic package including lifestock from their ancestral Anatolian homelands. The second migration witnessed the rapid expansion of Western Steppe Herder (WSH) related ancestry [29, 30].

In our regional reconstruction (Fig. S6), the expansion of farmers from Southwest Asia to Europe began with a decline of high population densities in Anatolia and lower densities in the pre-Neolithic Black Sea region around 8.4–8.3 ka BP, concomitant with a boom in Greece and the Balkans as indicative of a westward movement of early farmers across the Aegean Sea [31, 32]. This first migration wave is contemporaneous with a growth boom in Western Europe around 8–7.9 ka BP, likely also involving hunter-gatherer societies since farming is for that time documented only for South Europe. In Southwest Europe, the demographic boom persisted throughout much of the 8th millennium BP, thus pointing to a successful adaptation of early farmers around the Mediterranean. With the onset of farming in Central Europe 7.4–7.2 ka BP, positive population growth regained a continental momentum (Tab. S3). The spatial structure of the growth pattern matches our current understanding that farming progressed into Europe along two separate streams, a continental one and a coastal one propagating along the Mediterranean coast, which reached Central and western Central Europe between 7.5 and 7.2 ka BP [33, 34]. One millennium later, farming arrived on the British Isles [35], and after some further delay in most of Scandinavia [36, 37]. During this period continental wide trade and prestige networks are witnessed by the long range distribution and use of early copper tools in Eastern Europe and Jade axes in the West [38, 39].

The second migration wave involving diverse groups sharing WSH ancestries spatially extended farming. The reconstructed growth peak at 4.85 ka BP, resulting from a continental alignment of regional growth rates (Fig. S6), coincides with the onset of the Corded Ware Culture in Northeast Europe [40]. In Western and Central Europe, this phase is associated with broadly defined cultural units such as the Bell Beaker phenomenon (see also Tab. S3) and Únětice [29].

The expansion of farming technologies did not progress continuously across Europe (nor did it elsewhere) but rather as rapid shifts after stagnation periods [41–44]. Our analysis suggests that the spread was accelerated in periods of stable environmental conditions or, vice versa, slowed down at low climate stability (at 7.8–7.5 and 7–6.8 ka BP). Also, preceding phases of low stability (8.5–8.3, 7.8–7.5, and 5.3–5.1 ka BP) together with negative peaks in RGR may have paved the way for immigrants, as already proposed for the period before the establishment of WSH ancestries in central and northern Europe [45]. Our study adds to the discussion on the role of climate in this pattern [42, 46], as expansions coincided with periods of greater climate stability.

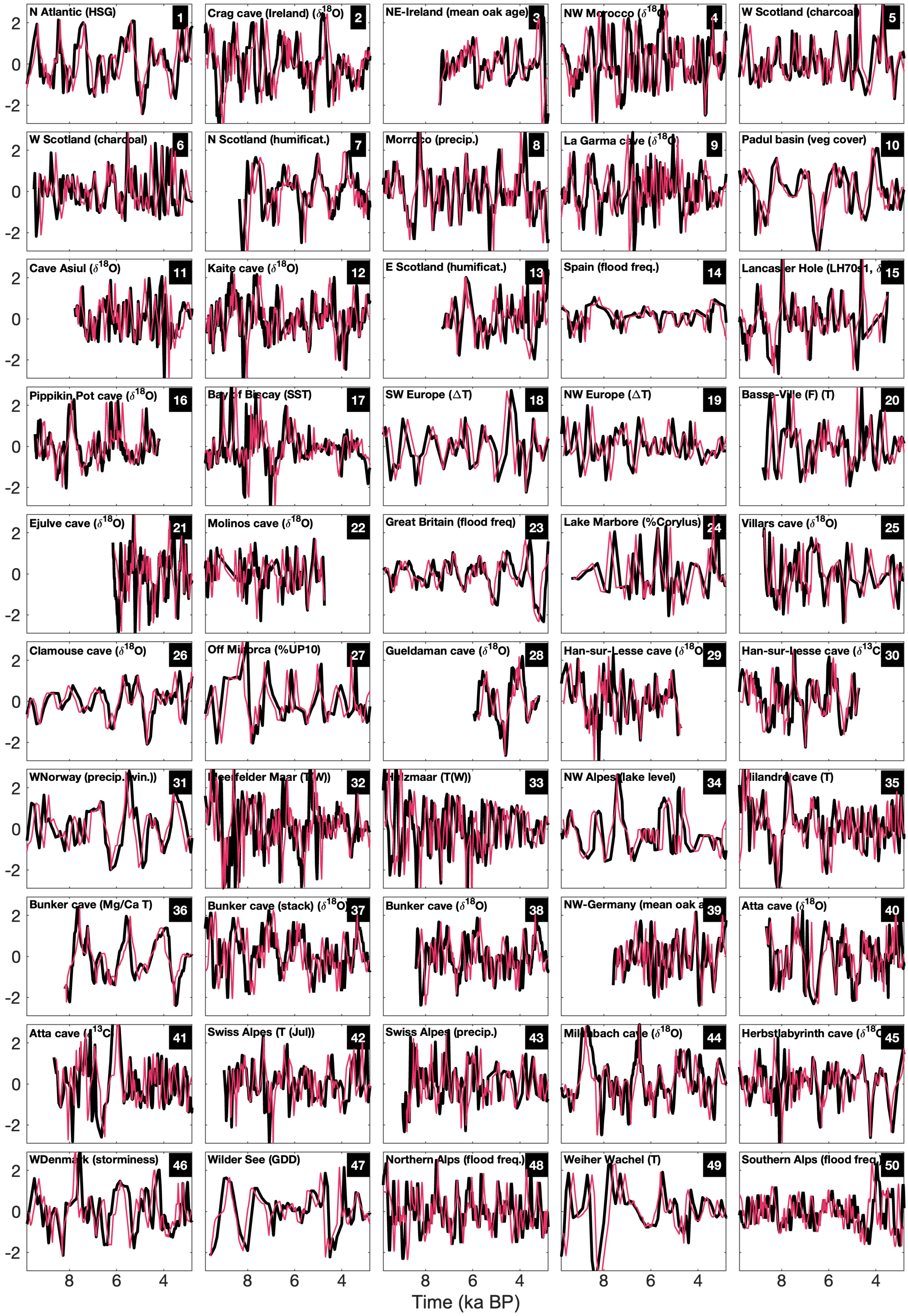
N Atlantic (HSG)
Crag cave (Ireland) (δ18O)
NE-Ireland (mean oak age)
NW Morocco (δ18O)
W Scotland (charcoal)
W Scotland (charcoal)
N Scotland (humificat.)
Morroco (precip.)
La Garma cave (δ18O)
Padul basin (veg cover)
Cave Asiul (δ18O)
Kaite cave (δ18O)
E Scotland (humificat.)
Spain (flood freq.)
Lancaster Hole (LH70s1,
Pippikin Pot cave (δ18O)
Bay of Biscay (SST)
SW Europe (ΔT)
NW Europe (ΔT)
Basse-Ville (F) (T)
Ejulve cave (δ18O)
Molinos cave (δ18O)
Great Britain (flood freq)
Lake Marbore (%Corylus)
Villars cave (δ18O)
Clamouse cave (δ18O)
Off Minorca (%UP10)
Gueldaman cave (δ18O)
Han-sur-Lesse cave (δ18O
Han-sur-Lesse cave (δ13C
WNorway (precip. win.))
Meerfelder Maar (T(W))
Holzmaar (T(W))
NW Alpes (lake level)
Milandre cave (T)
Bunker cave (Mg/Ca T)
Bunker cave (stack) (δ18O)
Bunker cave (δ18O)
NW-Germany (mean oak a
Atta cave (δ18O)
Atta cave (δ13C
Swiss Alpes (T (Jul))
Swiss Alpes (precip.)
Milchbach cave (δ18O)
Herbstlabyrinth cave (δ18O
WDenmark (storminess)
Wilder See (GDD)
Northern Alps (flood freq.)
Weiher Wachel (T)
Southern Alps (flood freq.
1
2
3
4
5
6
7
8
9
10
11
12
13
14
15
16
17
18
19
20
21
22
23
24
25
26
27
28
29
30
31
32
33
34
35
36
37
38
39
40
41
42
43
44
45
46
47
48
49
50
2
0
-2
8
6
4
Time (ka BP)

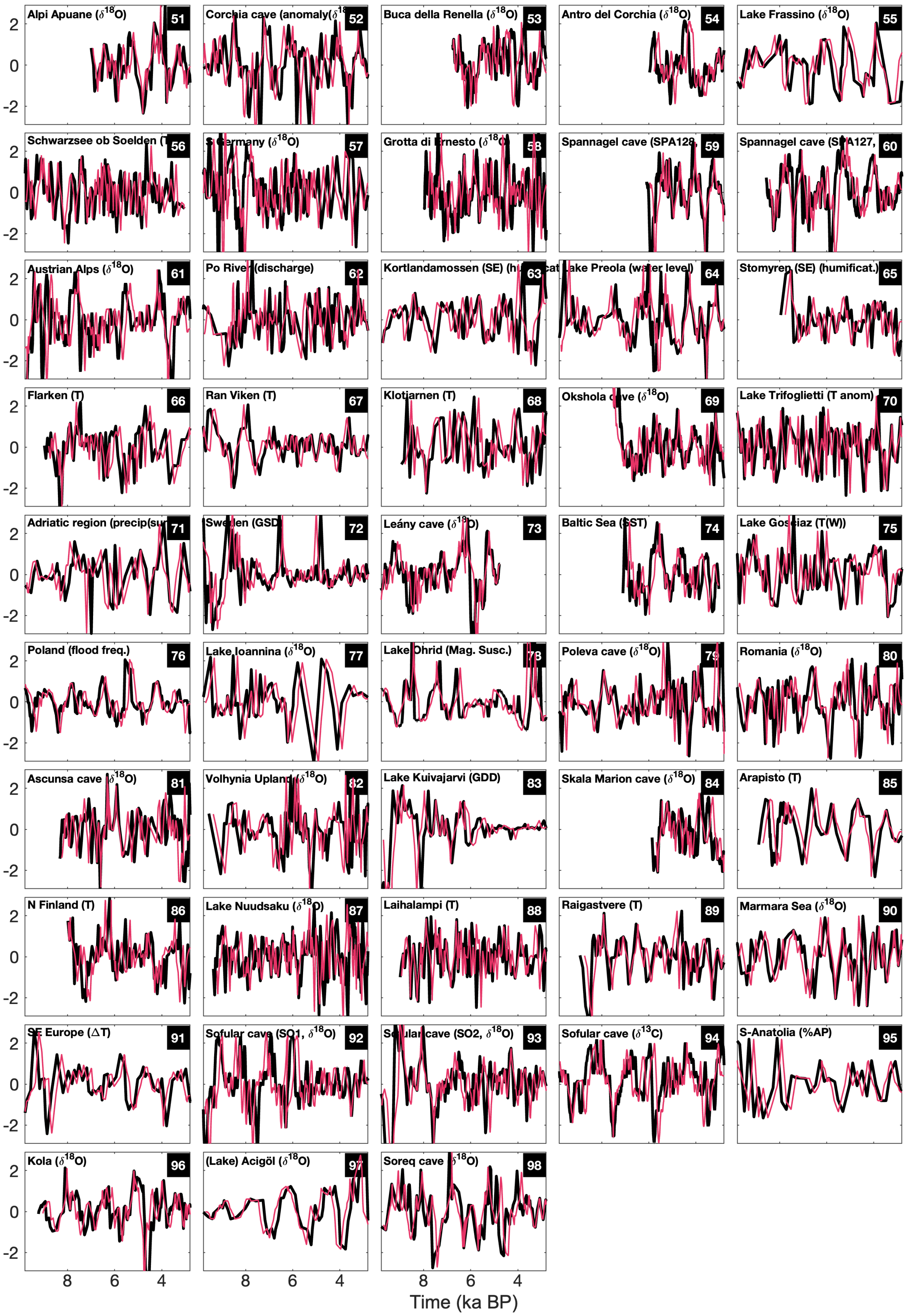


**Fig. S1** Normalized paleoclimate proxies (Tab. S2) in or around Europe before (black line) and after (red) application of dynamic time warping.

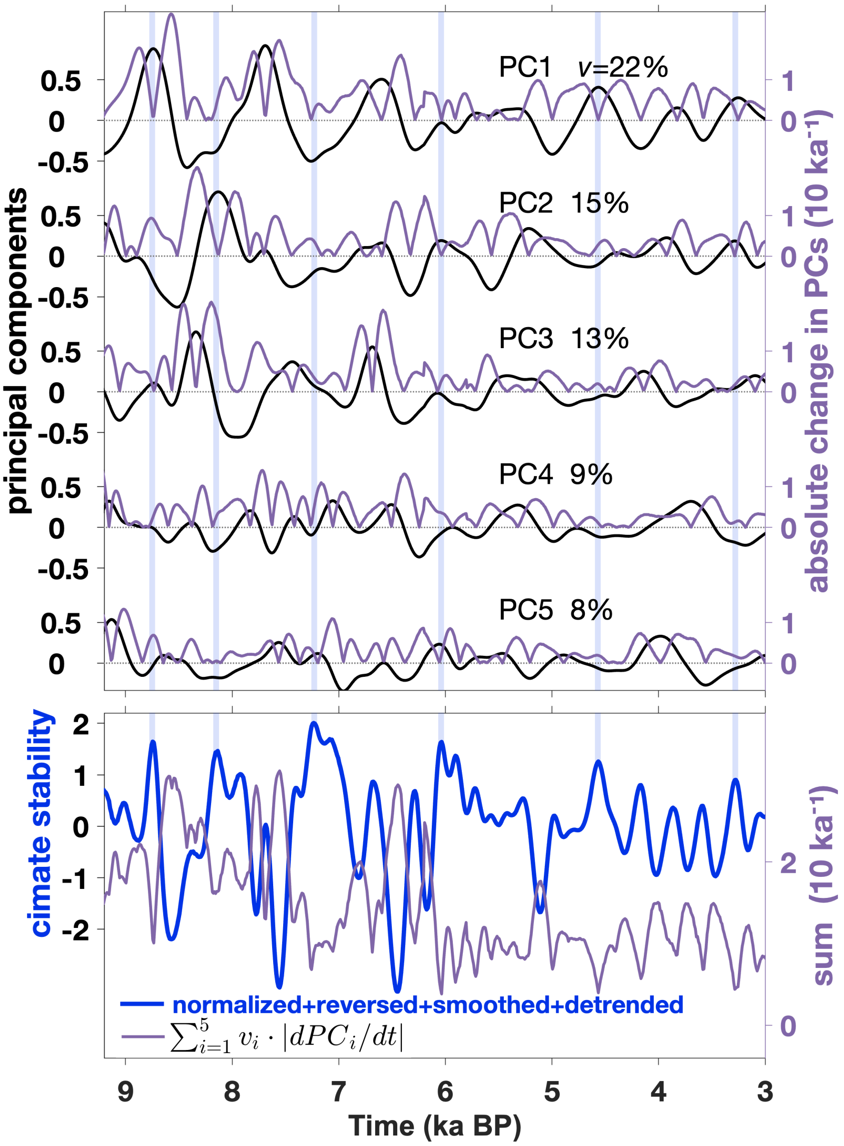


**Fig. S2** Construction of an index for climate stability. Top: From the principal component analysis (PCA) of 98 paleoclimate time-series (Tab. S2, Fig. S1) the first five PCs (black lines) are shown together with the respective explained variance $v_i$ and the absolute values of temporal derivatives (blue-purple lines). Bottom: The weighed sum of absolute temporal changes ($\sum_{i=1}^{5} v_i \cdot |dPC_i/dt|$) is normalized, reversed in sign, band-passed filtered, thus smoothed and de-trended (see Methods) to yield an estimate for climate stability: low values of this index indicate periods where the PCs are stagnant - including at extreme values - as marked by light blue bars, while high values indicate periods of fast changes in PCs.

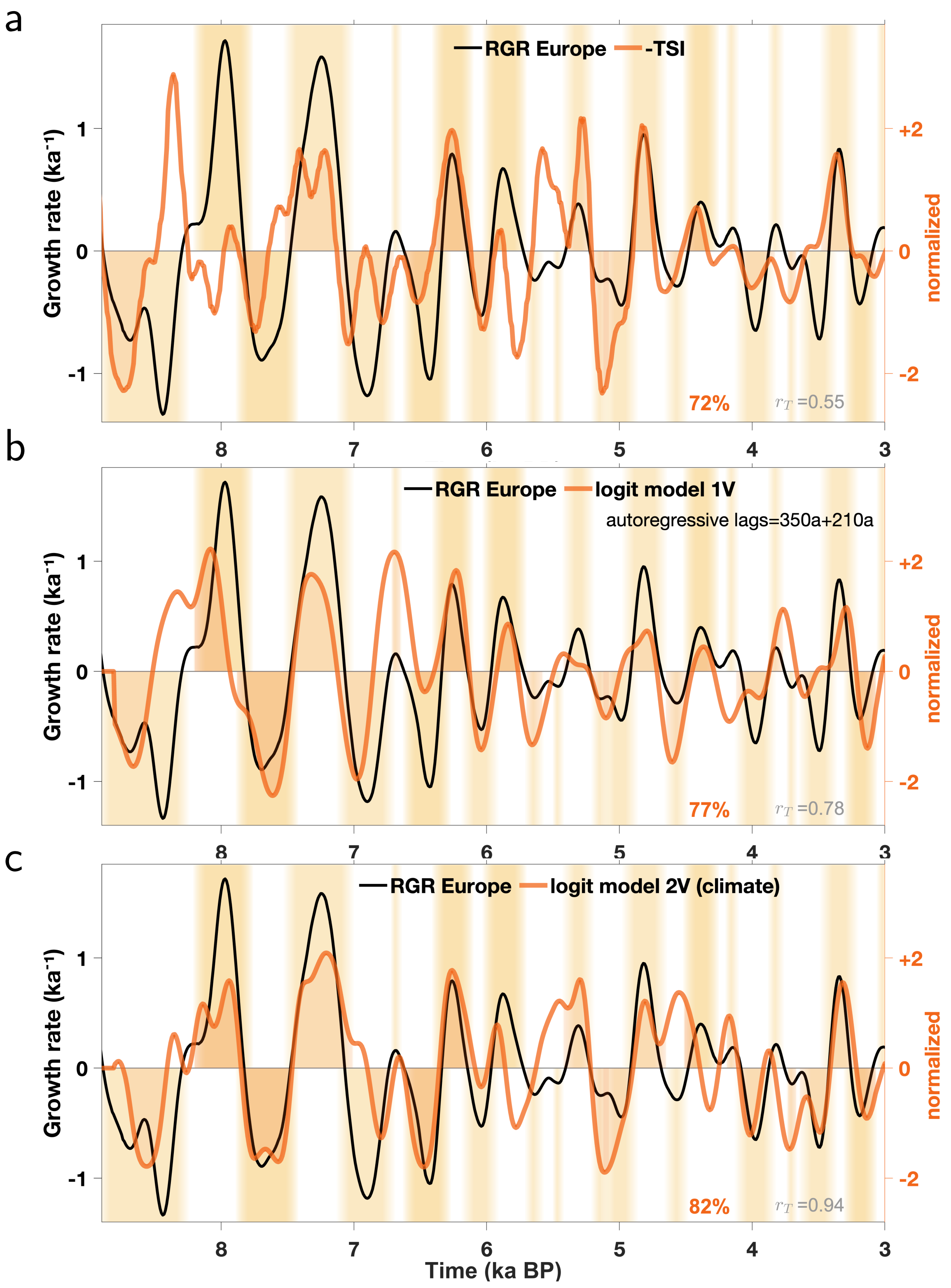


**Fig. S3** European RGR compared to the explanatory variables (a) negative total solar irradiance (TSI), (b) preceding RGR ('1V' logit model), and (c) the '2V' logit model with PC-based climate stability and TSI as input. Negative and positive growth phases (beige shading) underlay the calculation of the phase overlap (orange shading), which is given in bold orange numbers together with the trim correlation $r_T$ in light grey. Here and in the following, the compared time-series is normalized (de-trended and division by standard deviation $\sigma$) and plotted as red (or reddish) line.

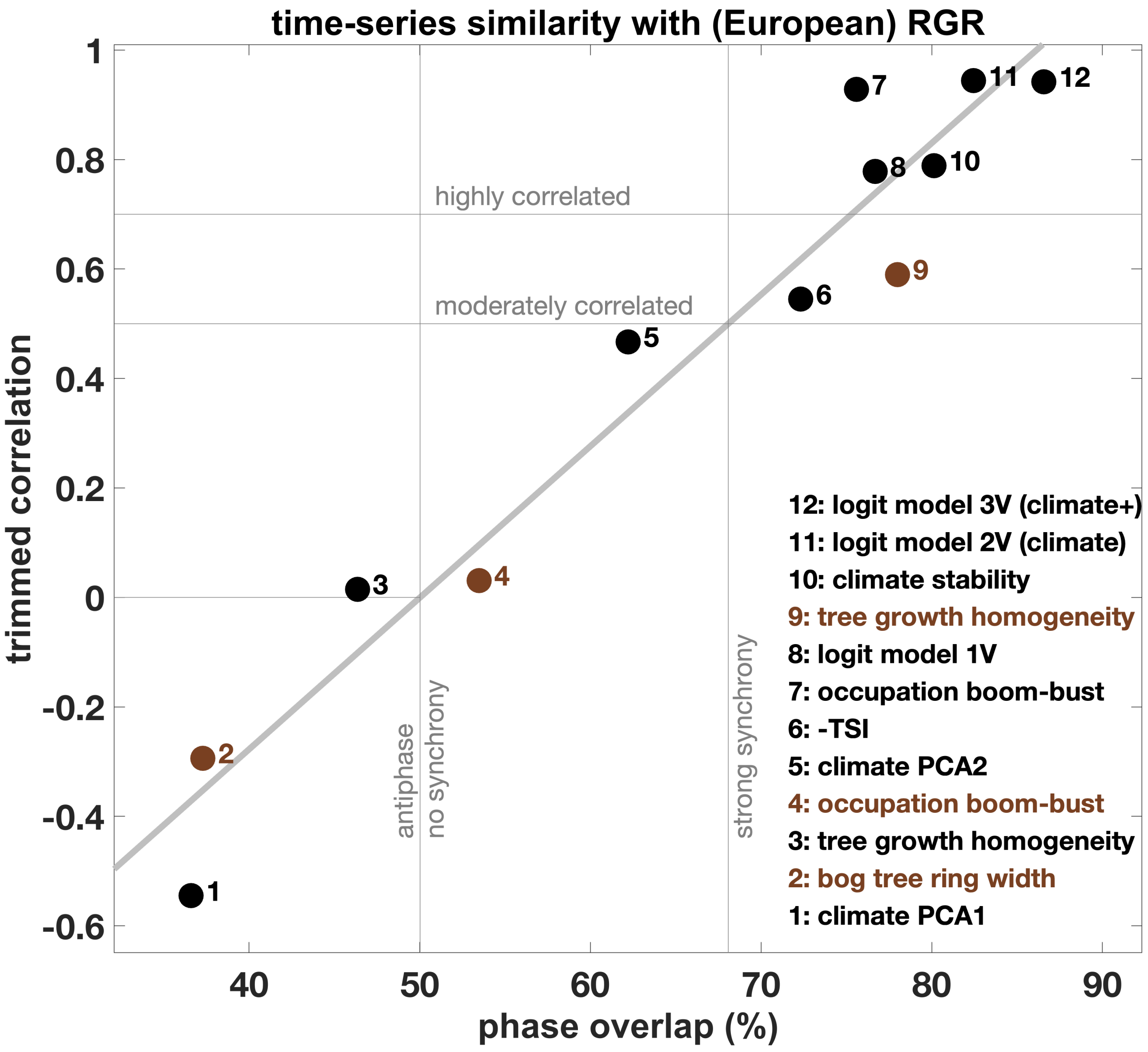


**Fig. S4** Trimmed correlation $r_{\mathrm{T}}$ and phase overlap as statistical similarity measures for comparing mostly climate related time-series to variations in (European) RGR. Black circles describe the relation to the reference European RGR (non-normalized calibration, based on regional resolution), brown circles the one to other RGR reconstructions: 4, RGR with normalized calibration; 2, 9, RGR of N Ireland.

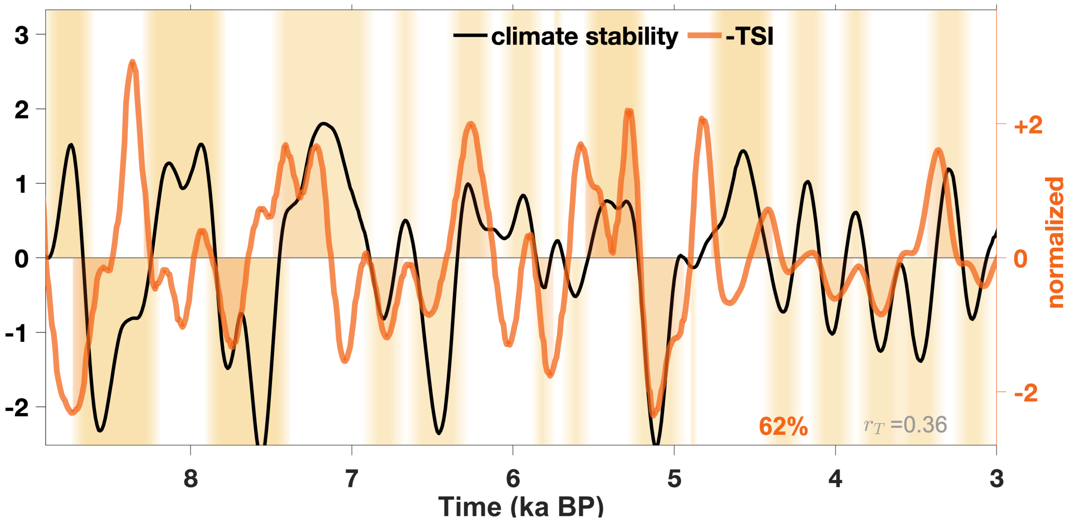


**Fig. S5** Fluctuations in European climate stability and solar forcing (TSI), both in normalized units.

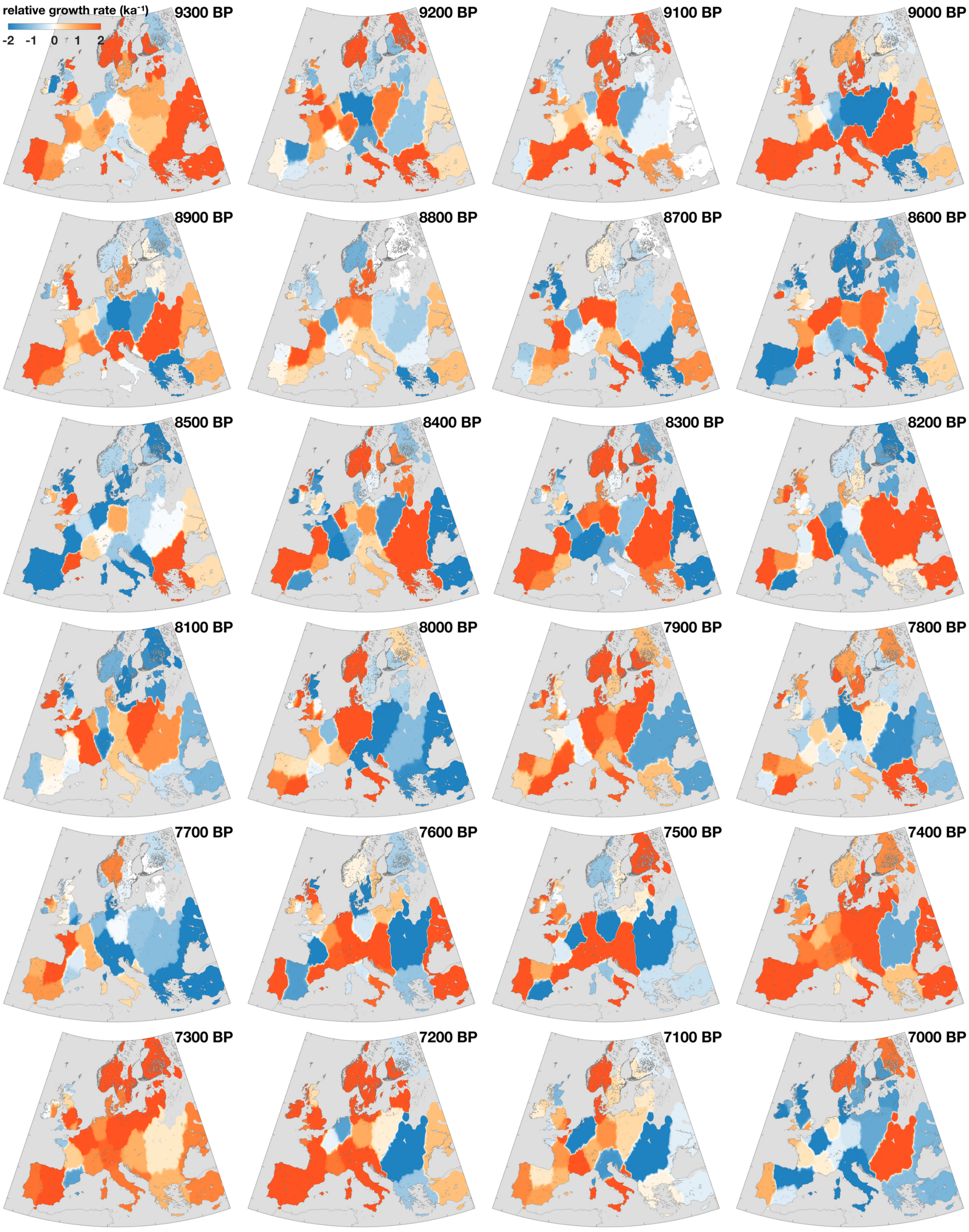
relative growth rate (ka⁻¹)
-2 -1 0 1 2
9300 BP
9200 BP
9100 BP
9000 BP
8900 BP
8800 BP
8700 BP
8600 BP
8500 BP
8400 BP
8300 BP
8200 BP
8100 BP
8000 BP
7900 BP
7800 BP
7700 BP
7600 BP
7500 BP
7400 BP
7300 BP
7200 BP
7100 BP
7000 BP

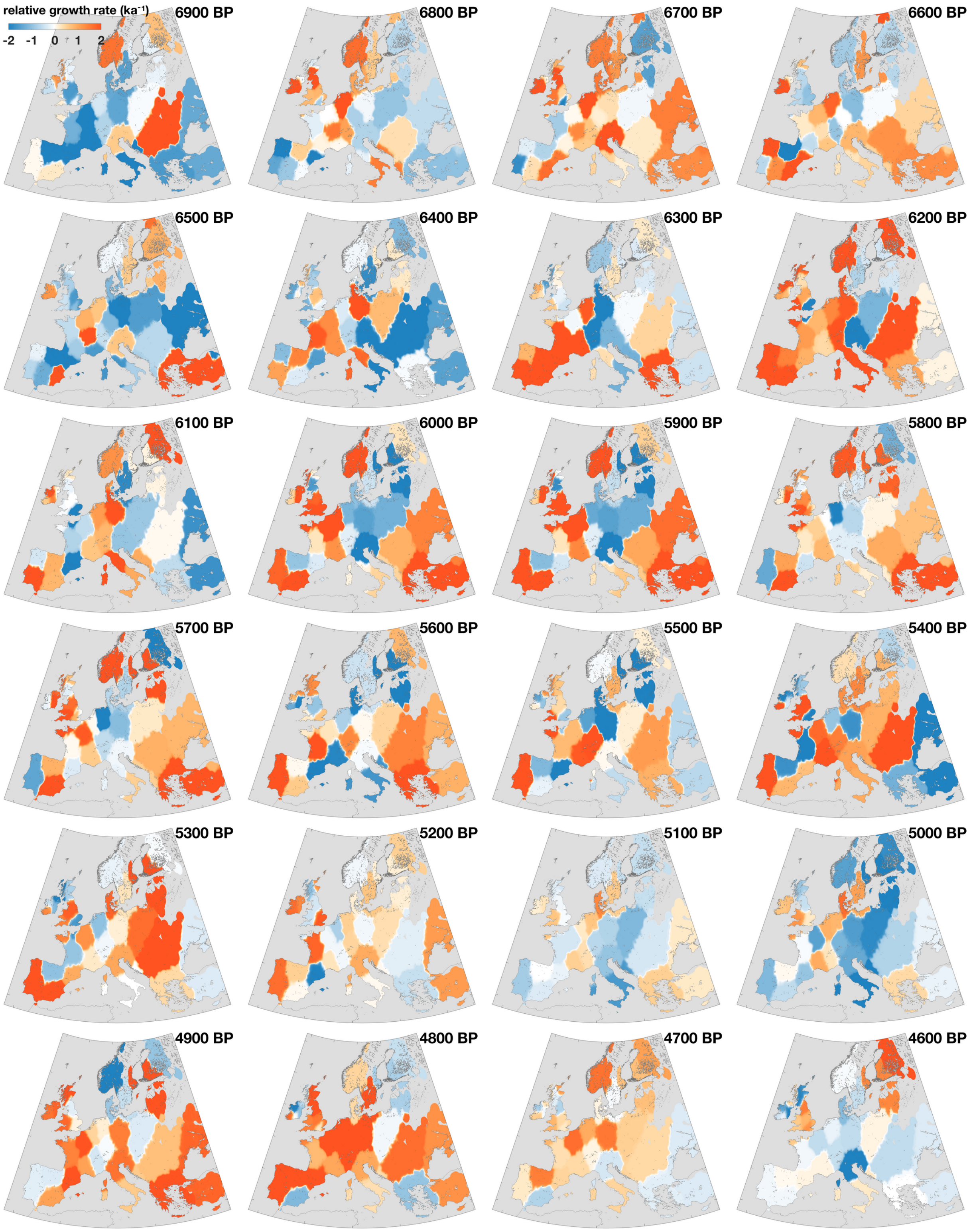
relative growth rate (ka⁻¹)
-2 -1 0 1 2
6900 BP
6800 BP
6700 BP
6600 BP
6500 BP
6400 BP
6300 BP
6200 BP
6100 BP
6000 BP
5900 BP
5800 BP
5700 BP
5600 BP
5500 BP
5400 BP
5300 BP
5200 BP
5100 BP
5000 BP
4900 BP
4800 BP
4700 BP
4600 BP

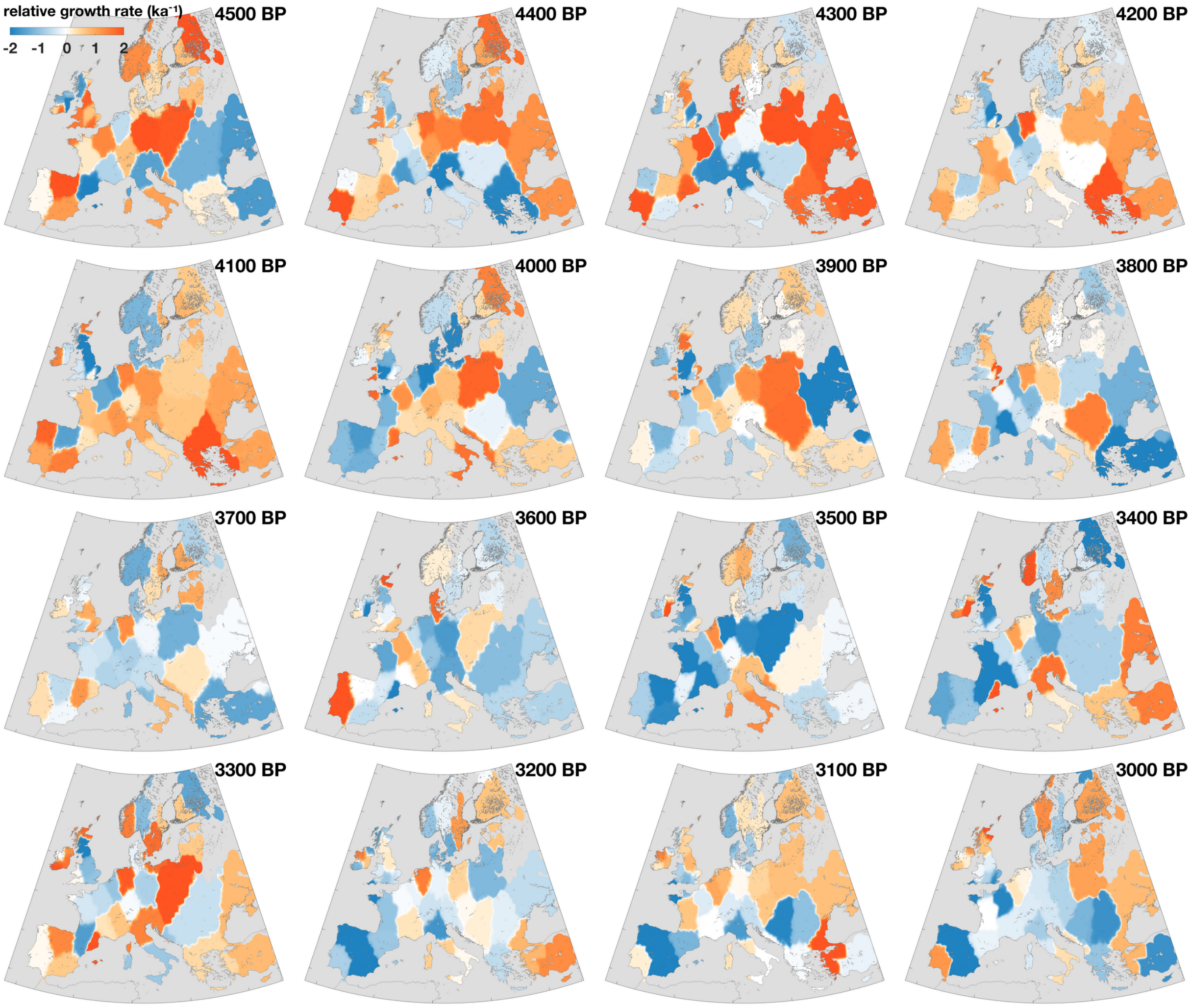


**Fig. S6** Time–slice maps of regional population growth rates in Europe based on changes in SPDs indicating population booms (orange areas) and busts (blue). Note the flexible region layout changing every 400 a.

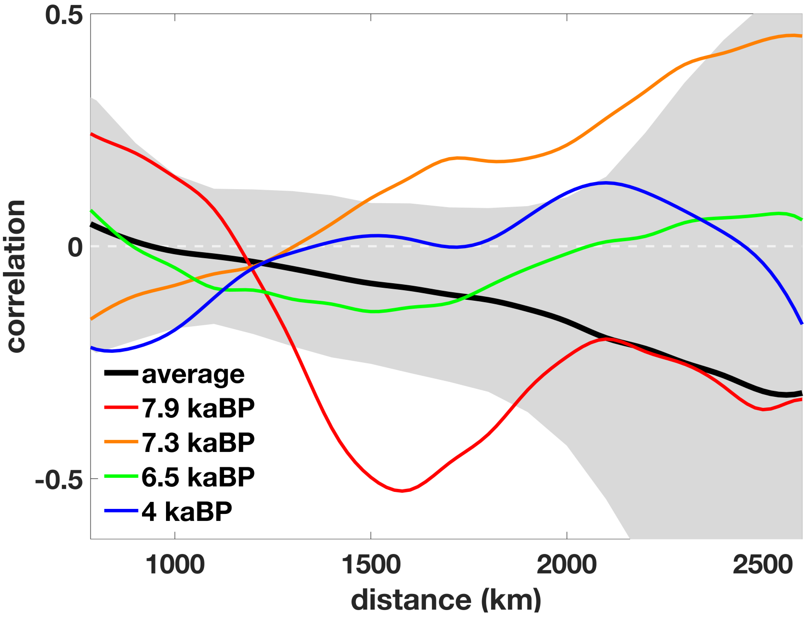


**Fig. S7** Spatial autocorrelation of $^{14}C$-based estimates for population growth (RGR) in Europe (see Fig. S6). The average for the Holocene (black line with standard deviation as grey shading) is compared to the spatial correlation during individual periods marking either booms (7.9 and 7.3 kaBP) or busts (6.5 and 4 kaBP). The range of distances is bounded by upper cluster size of the SPD approach and about half the continental extension.

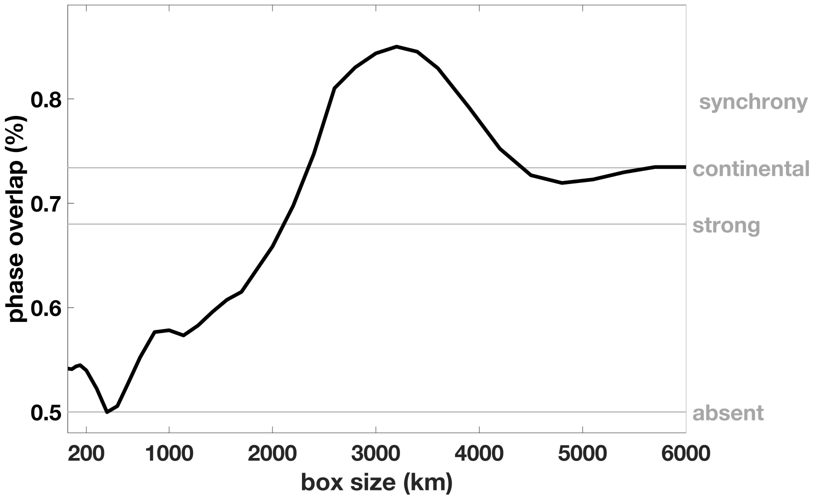


**Fig. S8** Scale dependence of synchrony between population growth (RGR) and solar forcing (-TSI). Phase overlap of negative TSI with RGR averaged over a box around Northern Ireland is shown for increasing box scale.

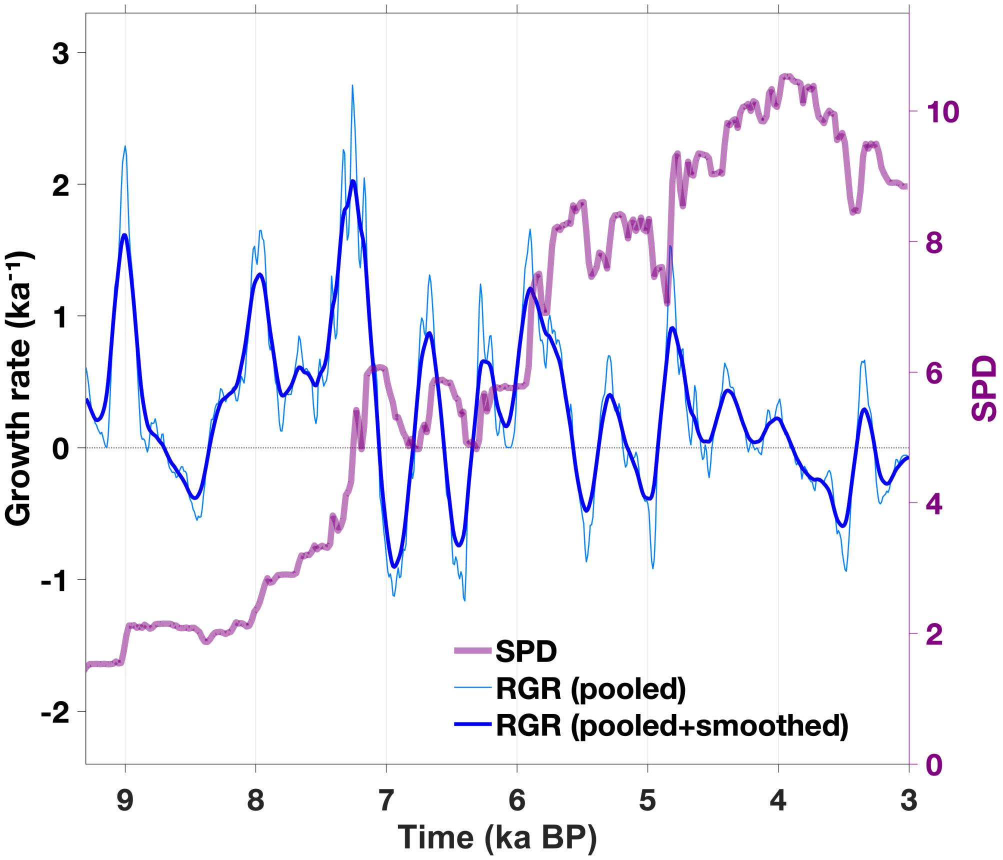


**Fig. S9** Summed probability distributions (SPD) of pooled European $^{14}$C dates (pink line) and corresponding relative changes of rate (RGR), which were smoothed using a filter along a time window of 30 a (thin blue line) and 130 a (thick blue line), respectively. Note that due to division by SPD in the RGR calculation, small fluctuations during the first millennia in the Holocene lead to relatively large excursions in RGR.

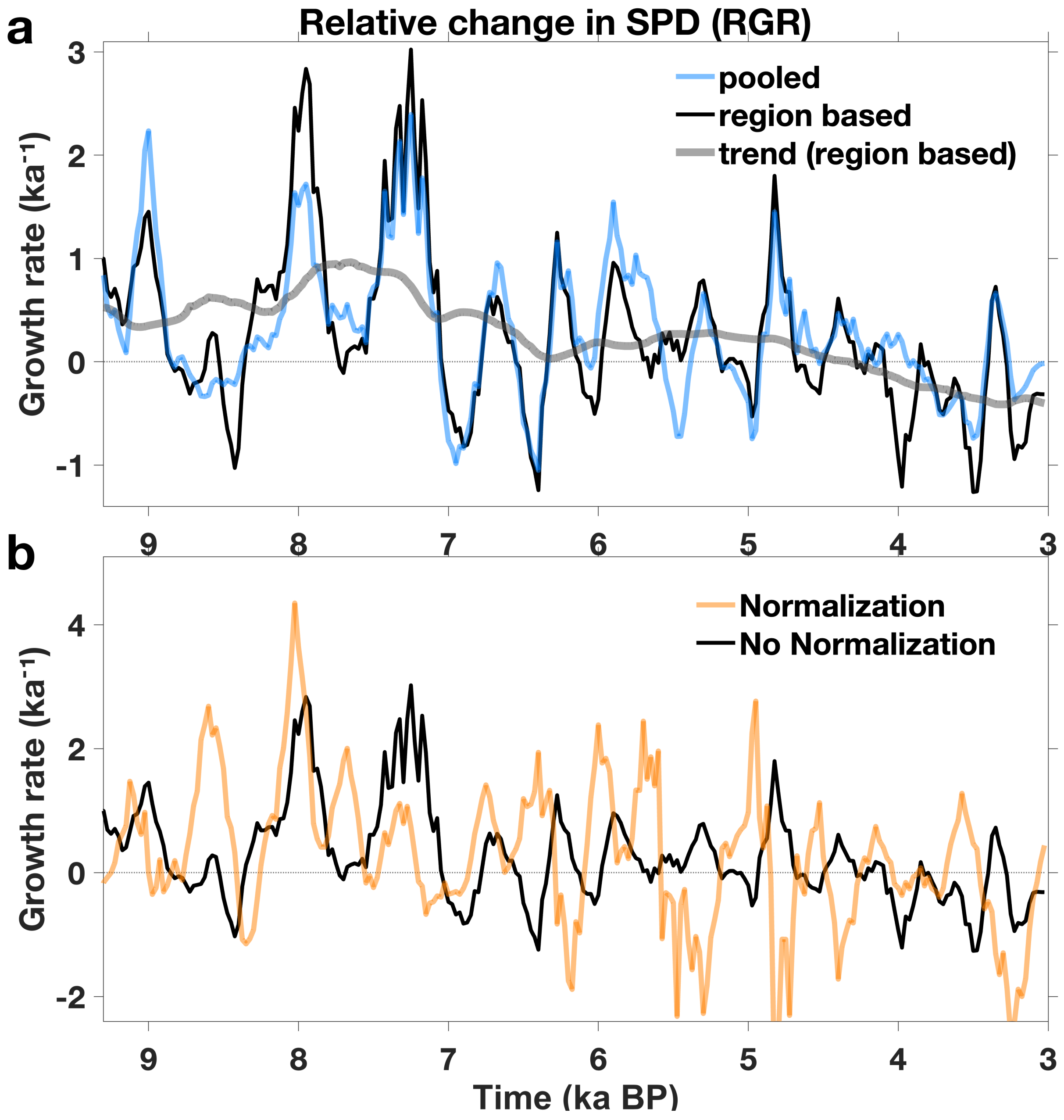


**Fig. S10** Comparison of European relative growth rates (RGR) based on SPDs calculated using different approaches: a, The reference non-normalized method of region based averaging (black line, with 1.5 ka moving average in grey) vs. the pooled non-normalized method without spatial resolution (blue). b, RGR based on reference non-normalized SPD in black vs. RGR based on normalized SPD in orange.

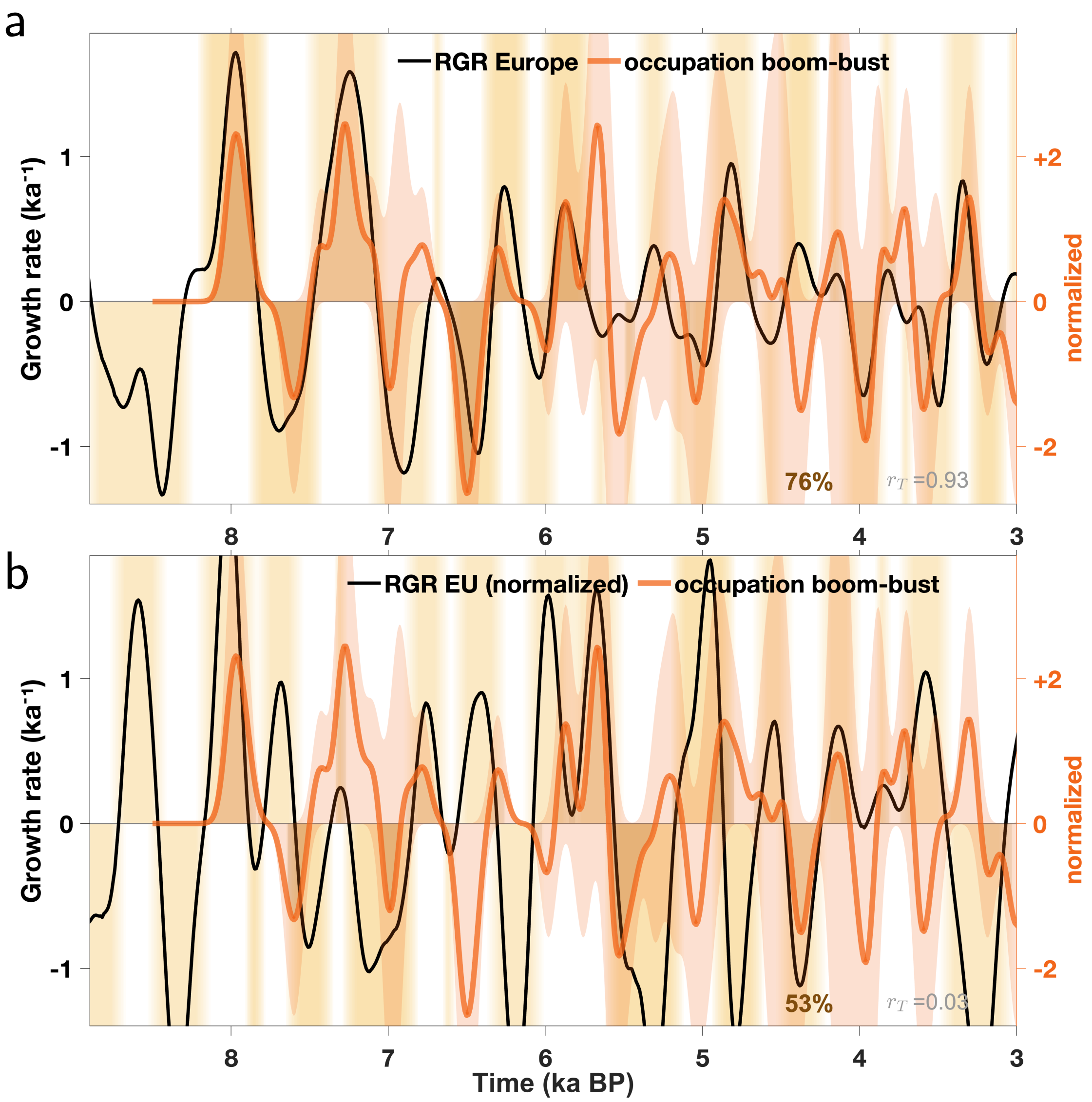


**Fig. S11** SPD-based RGR vs. the difference between the density of booms and the density of busts. The underlying densities of booms and busts, resp., originate from occupation data and are outlined by red shading. a, RGR with non-normalized calibration (reference). b, RGR with normalized calibration. For phase shading and normalized units see Fig. S3.

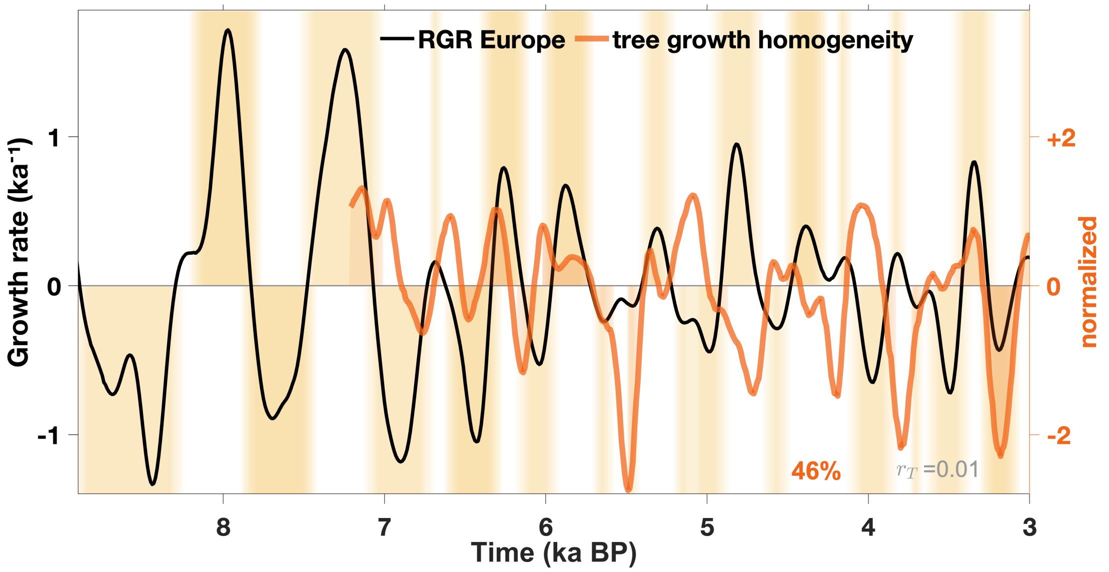


**Fig. S12** Continental RGR compared to negative normalized standard deviation of tree ring width in Irish bogs as a local proxy for climate stability and homogeneity.

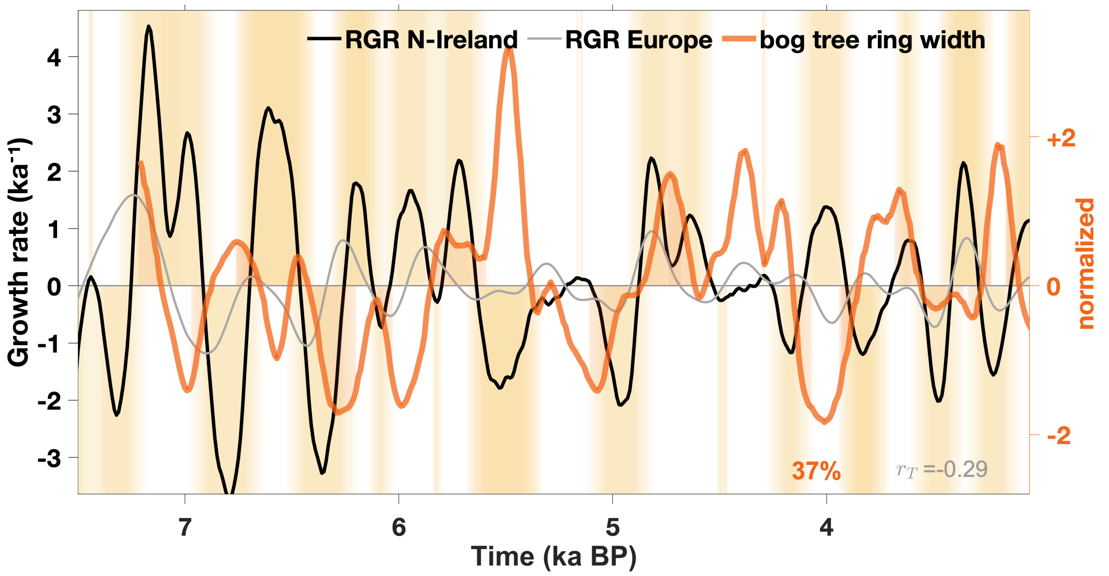


**Fig. S13** Northern Ireland RGR compared to tree ring width in Irish bogs as a local proxy for climatic suitability of tree growth. The European growth rate is shown for comparison as thin grey line.

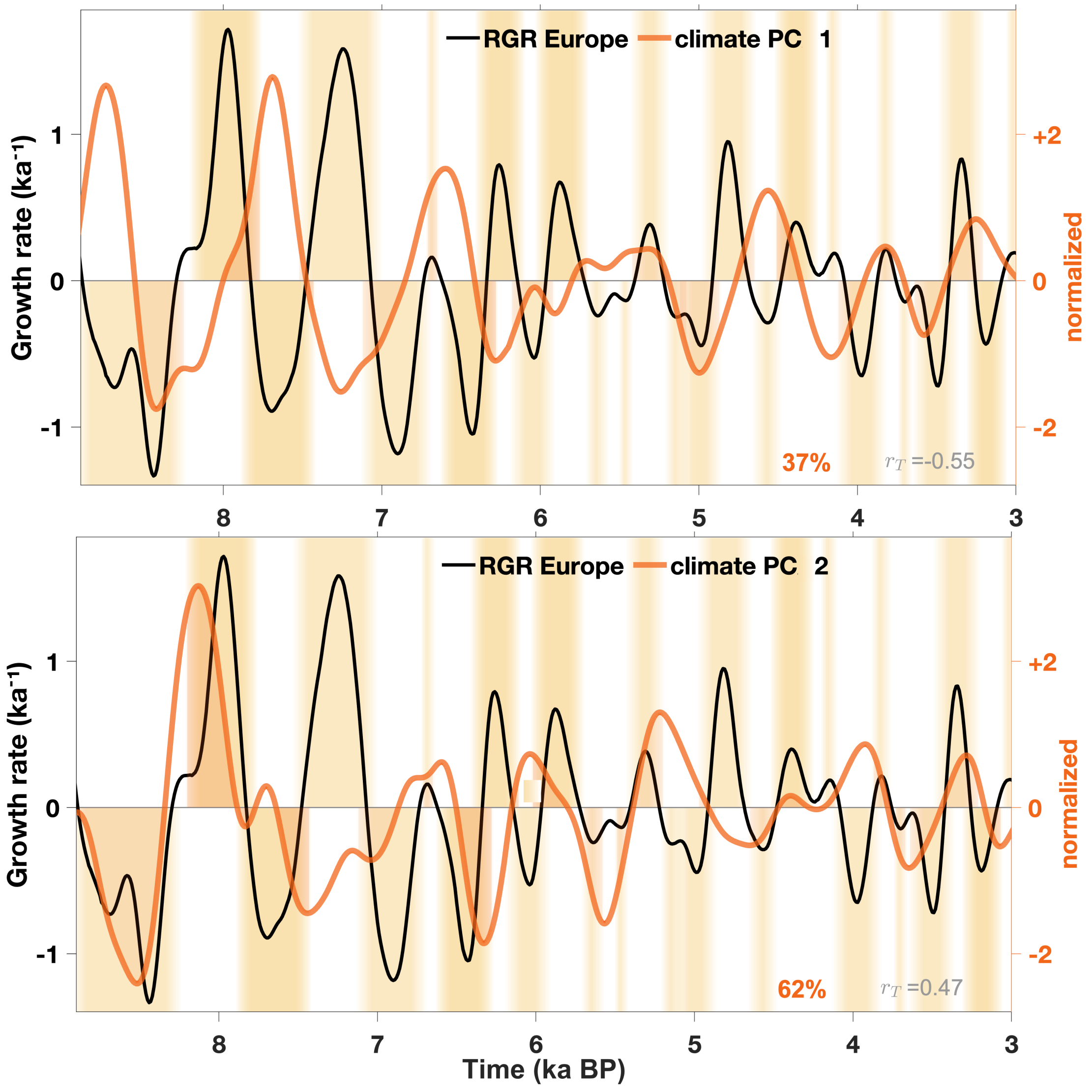


**Fig. S14** European RGR contrasted to the first and second principal component (PC) of 98 paleoclimate proxies.

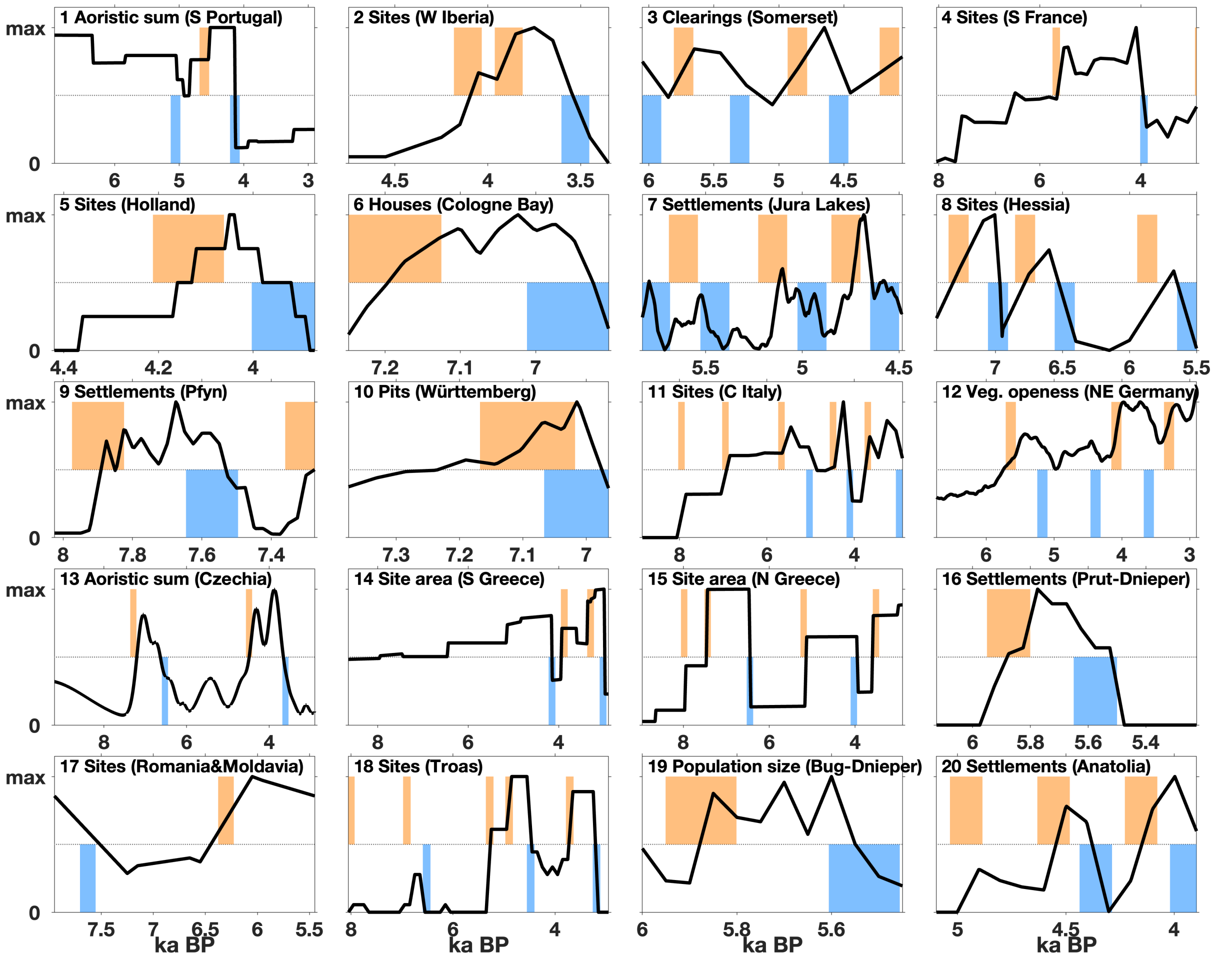


**Fig. S15** Time-series of alternative proxies for population density from the archeological literature (see Tab. S4). Booms (red shading) or busts (blue shading) were attributed if change rates exceeded a critical threshold. Shadings for booms and busts appear with non-uniform widths due to the different lengths of time segments.

**Table S1** Collection of $^{14}C$ datasets as basis of the demography reconstruction for Europe.

| Name | Date | URL (https://) | Ref. |
|---|---|---|---|
| AGRICHANGE | 2021-05-21 | | [47] |
| AIDA | 2021-09-08 | | [48] |
| BDA | 2020-03-29 | nakala.fr/10.34847/nkl.dde9fnm8 | [49] |
| c14bazAAR | 2021-09-08 | github.com/ropensci/c14bazAAR | [50] |
| CALPAL | 2020-08-20 | uni-koeln.academia.edu/BernhardWeninger/Cal | [51] |
| CONTEXT | 2016-09-26 | context-database.uni-koeln.de | - |
| EUBAR | 2017-10-02 | telearchaeology.org//EUBAR | [52] |
| EUROEVOL | 2015-07-09 | github.com/ahb108/rcarbon | [53–55] |
| IRDD | 2018-08-13 | zenodo.org/record/3367518 | [56] |
| Katsianis et al. | 2020-08-20 | doi.org/10.5522/04/12489137.v1 | [57] |
| MEDAFRICARBON | 2020-03-20 | zenodo.org/record/3689716 | [58] |
| dASIS | 2021-09-08 | dasis.dainst.org/ | [59] |
| NERD | 2021-09-08 | zenodo.org/record/576786 | [60] |
| PACEA | 2020-01-22 | | [61] |
| Palmisano et al. | 2017-09-23 | zenodo.org/record/5846835 | [62] |
| p3k14c | 2022-09-21 | github.com/people3k/p3k14c | [63] |
| RADON | 2021-09-08 | radon.ufg.uni-kiel.de | [55] |
| RADONb | 2021-09-08 | radonb.ufg.uni-kiel.de | [64] |

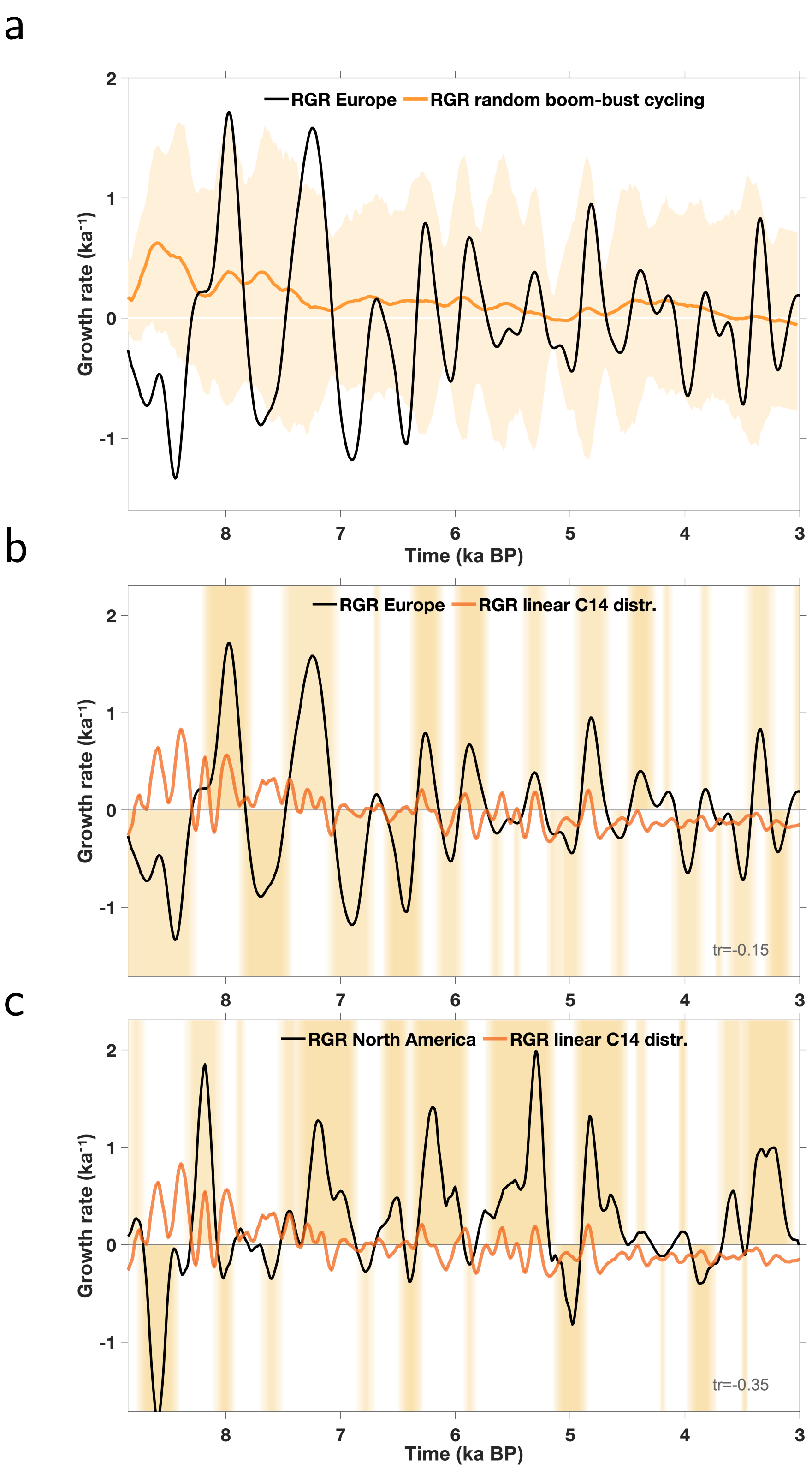


**Fig. S16** a, Mean of $5{\cdot}10^5$ growth trajectories, which share a similar boom and bust number and duration with the reference RGR reconstruction (see Methods), while exact timings are shifted randomly. Color shading: 25% and 75% percentiles. European RGR is plotted as black line. b, European RGR compared to the RGR of a linearly increasing $^{14}$C distribution (red line). Non-zero values represent the artificial effect of variable slopes of the calibration curve on SPDs. Color shading: boom and bust phases. c, Analogue comparison for RGR of North America.

**Table S2** Location, type, and reference of climate proxy time-series (shown in Fig. S1). Numbers ordered from West to East identify the position in the map (Fig. 1). 'anomaly' in $\delta^{18}O$, Mg/Ca, and $\delta^{13}C$.

| No | Site | Proxy | Ref. | No | Site | Proxy | Ref. |
|---|---|---|---|---|---|---|---|
| 1 | N Atlantic | HSG | [65] | 2 | Crag cave (Ireland) | $\delta^{18}O$ | [66] |
| 3 | NE-Ireland | mean oak age | [67] | 4 | NW Morocco | $\delta^{18}O$ | [68] |
| 5 | W Scotland | charcoal | [69] | 6 | W Scotland | charcoal | [69] |
| 7 | N Scotland | humificat. | [69] | 8 | Morroco | precip. | [70] |
| 9 | La Garma cave | $\delta^{18}O$ | [71] | 10 | Padul basin | veg cover | [72] |
| 11 | Cave Asiul | $\delta^{18}O$ | [73] | 12 | Kaite cave | $\delta^{18}O$ | [74] |
| 13 | E Scotland | humificat. | [75] | 14 | Spain | flood freq. | [76] |
| 15 | Lancaster Hole (LH70s1) | $\delta^{18}O$ | [77] | 16 | Pippikin Pot cave | $\delta^{18}O$ | [78] |
| 17 | Bay of Biscay | SST | [79] | 18 | SW Europe | $\Delta T$ | [80] |
| 19 | NW Europe | $\Delta T$ | [80] | 20 | Basse-Ville (F) | T | [81] |
| 21 | Ejulve cave | $\delta^{18}O$ | [82] | 22 | Molinos cave | $\delta^{18}O$ | [82] |
| 23 | Great Britain | flood freq | [83] | 24 | Lake Marbore | %Corylus | [84] |
| 25 | Villars cave | $\delta^{18}O$ | [85] | 26 | Clamouse cave | $\delta^{18}O$ | [86] |
| 27 | Off Minorca | %UP10 | [87] | 28 | Gueldaman cave | $\delta^{18}O$ | [88] |
| 29 | Han-sur-Lesse cave | $\delta^{18}O$ | [89] | 30 | Han-sur-Lesse cave | $\delta^{13}C$ | [89] |
| 31 | WNorway | precip.(win.) | [90] | 32 | Meerfelder Maar | T(W) | [91] |
| 33 | Holzmaar | T(W) | [91] | 34 | NW Alpes | lake level | [92] |
| 35 | Milandre cave | T | [93] | 36 | Bunker cave | Mg/Ca T | [94] |
| 37 | Bunker cave (stack) | $\delta^{18}O$ | [94] | 38 | Bunker cave | $\delta^{18}O$ | [94] |
| 39 | NW-Germany | mean oak age | [67] | 40 | Atta cave | $\delta^{18}O$ | [95] |
| 41 | Atta cave | $\delta^{13}C$ | [95] | 42 | Swiss Alpes | T (Jul) | [96] |
| 43 | Swiss Alpes | precip. | [96] | 44 | Milchbach cave | $\delta^{18}O$ | [97] |
| 45 | Herbstlabyrinth cave | $\delta^{18}O$ | [98] | 46 | WDenmark | storminess | [99] |
| 47 | Wilder See | GDD | [81] | 48 | Northern Alps | flood freq. | [16] |
| 49 | Weiher Wachel | T | [81] | 50 | Southern Alps | flood freq. | [16] |
| 51 | Alpi Apuane | $\delta^{18}O$ | [100] | 52 | Corchia cave | anomaly | [101] |
| 53 | Buca della Renella | $\delta^{18}O$ | [100] | 54 | Antro del Corchia | $\delta^{18}O$ | [102] |
| 55 | Lake Frassino | $\delta^{18}O$ | [103] | 56 | Schwarzsee ob Soelden | T(S) | [104] |
| 57 | S Germany | $\delta^{18}O$ | [105] | 58 | Grotta di Ernesto | $\delta^{18}O$ | [106] |
| 59 | Spannagel cave (SPA128) | $\delta^{18}O$ | [107] | 60 | Spannagel cave (SPA127) | $\delta^{18}O$ | [107] |
| 61 | Austrian Alps | $\delta^{18}O$ | [108] | 62 | Po River | discharge | [109] |
| 63 | Kortlandamossen (SE) | humificat. | [110] | 64 | Lake Preola | water level | [111] |
| 65 | Stomyren (SE) | humificat. | [110] | 66 | Flarken | T | [112] |
| 67 | Ran Viken | T | [81] | 68 | Klotjarnen | T | [113] |
| 69 | Okshola cave | $\delta^{18}O$ | [114] | 70 | Lake Trifoglietti | T anom | [115] |
| 71 | Adriatic region | precip(sum.) | [109] | 72 | Sweden | GSD | [116] |
| 73 | Leány cave | $\delta^{18}O$ | [117] | 74 | Baltic Sea | SST | [118] |
| 75 | Lake Gosciaz | T(W) | [81] | 76 | Poland | flood freq. | [76] |
| 77 | Lake Ioannina | $\delta^{18}O$ | [119] | 78 | Lake Ohrid | Mag. Susc. | [120] |
| 79 | Poleva cave | $\delta^{18}O$ | [121] | 80 | Romania | $\delta^{18}O$ | [122] |
| 81 | Ascunsa cave | $\delta^{18}O$ | [123] | 82 | Volhynia Upland | $\delta^{18}O$ | [124] |
| 83 | Lake Kuivajarvi | GDD | [81] | 84 | Skala Marion cave | $\delta^{18}O$ | [125] |
| 85 | Arapisto | T | [126] | 86 | N Finland | T | [127] |
| 87 | Lake Nuudsaku | $\delta^{18}O$ | [128] | 88 | Laihalampi | T | [129] |
| 89 | Raigastvere | T | [130] | 90 | Marmara Sea | $\delta^{18}O$ | [131] |
| 91 | SE Europe | $\Delta T$ | [80] | 92 | Sofular cave (SO1) | $\delta^{18}O$ | [132] |
| 93 | Sofular cave (SO2) | $\delta^{18}O$ | [132] | 94 | Sofular cave | $\delta^{13}C$ | [132] |
| 95 | S-Anatolia | %AP | [133] | 96 | Kola | $\delta^{18}O$ | [134] |
| 97 | (Lake) Acigöl | $\delta^{18}O$ | [135] | 98 | Soreq cave | $\delta^{18}O$ | [136] |

**Table S3** Cultural phases linked to continental booms. Abbreviations: (C)entral, (N)orth, (S)outh, (E)ast, (W)est, (A)ll Europe, (MED)iterranean.

| Time (kaBP) | | Phase | Characteristic processes |
|---|---|---|---|
| 8.0 | LM | Late Mesolithic – Early Neolithic | Initial west-ward dispersal of early farming societies in SE and MED (Anatolian ancestry) |
| 7.3 | EN | Late Mesolithic – Early Neolithic | Early farming societies in C and MED |
| 6.7 | MN-I | Late Mesolithic – Middle Neolithic | Developed farming and late hunter-gatherer societies (Western Hunter-Gatherer ancestry) in N |
| 6.2 | MN-II | Middle Neolithic | Developed farming societies (W, SE); Initial dispersal of farming to N |
| 5.9 | MN-III | Middle Neolithic | Complex farming societies (C, N); Continued dispersal of farming to N |
| 5.3 | LN-I | Late Neolithic | Complex farming societies |
| 4.8 | LN-II | Late Neolithic | Westward dispersal of Corded Ware (WSH-related ancestry) |
| 4.4 | LN-III | Final Neolithic | Eastward dispersal of Bell Beaker |
| 3.8 | EBA | Early Bronze Age | Chiefdoms (N) and archaic state societies (SE) |
| 3.3 | MBA | Middle Bronze Age | Chiefdoms & early states; Consolidation of bronze metallurgy (C, N) |

**Table S4** Location, type, and reference of independent time series for proxies of population density. The geographic extent of sub-regions is plotted in Fig. 1.

| | Location/region | Proxy | Period (kaBP) | Ref. |
|---|---|---|---|---|
| 1 | S Portugal | Aoristic sum | 6.9–2.7 | [137] |
| 2 | W Iberia | Sites | 4.8–3.3 | [138] |
| 3 | Somerset | Clearings | 6.1–4.0 | [139] |
| 4 | S France | Sites | 8.0–0.5 | [140] |
| 5 | Holland | Sites | 4.4–3.9 | [141] |
| 6 | Cologne Bay | Houses | 7.3–6.9 | [142] |
| 7 | Jura Lakes | Settlements | 5.8–4.5 | [143] |
| 8 | Hessia | Sites | 6.2–5.5 | [144] |
| 9 | Pfyn | Settlements | 8.0–7.3 | [145] |
| 10 | Württemberg | Pits | 7.4–7.0 | [146] |
| 11 | C Italy | Sites | 8.9–2.9 | [147] |
| 12 | NE Germany | Veg. openess | 6.7–2.6 | [148] |
| 13 | Czechia | Aoristic sum | 10.0—0.0 | [149] |
| 14 | S Greece | Site area | 8.6–1.5 | [150] |
| 15 | N Greece | Site area | 9.0–1.6 | [150] |
| 16 | Prut-Dnieper | Settlements | 5.5–5.2 | [151] |
| 17 | Romania&Moldavia | Sites | 8.0–5.4 | [152] |
| 18 | Troas | Sites | 5.9–2.9 | [153] |
| 19 | Bug-Dnieper | Population size | 6.0–5.4 | [154, 155] |
| 20 | Anatolia | Settlements | 5.1–4.3 | [156] |

## Supplementary References


[1] Bamforth, D. B. & Grund, B. Radiocarbon calibration curves, summed probability distributions, and early Paleoindian population trends in North America. *J. Archaeol. Sci.* **39**, 1768–1774 (2012).

[2] Bleicher, N. Summed radiocarbon probability density functions cannot prove solar forcing of Central European lake-level changes. *Holocene* **23**, 755–765 (2013).

[3] Freeman, J. *et al.* Synchronization of energy consumption by human societies throughout the holocene. *Proc. Nat. Acad. Sci.* **115**, 9962–9967 (2018).

[4] Herschel, W. Observations tending to investigate the nature of the sun, in order to find the causes or symptoms of its variable emission of light and heat; with remarks on the use that may possibly be drawn from solar observations. *Phil. Trans. Roy. Soc.* **91**, 265–318 (1801).

[5] Eddy, J. A. The Maunder Minimum: The reign of Louis XIV appears to have been a time of real anomaly in the behavior of the sun. *Science* **192**, 1189–1202 (1976).

[6] Shindell, D. T., Schmidt, G. A., Mann, M. E., Rind, D. & Waple, A. Solar forcing of regional climate change during the Maunder Minimum. *Science* **294**, 2149–2152 (2001).

[7] Lohmann, G., Rimbu, N. & Dima, M. Climate signature of solar irradiance variations: analysis of long-term instrumental, historical, and proxy data. *Int. J. Climatology* **24**, 1045–1056 (2004).

[8] Baldwin, M. P. & Dunkerton, T. J. Propagation of the Arctic Oscillation from the stratosphere to the troposphere. *J. Geophys. Res. Atmo.* **104**, 30937–30946 (1999).

[9] Christiansen, B. A model study of the dynamical connection between the Arctic Oscillation and stratospheric vacillations. *J. Geophys. Res. Atmo.* **105**, 29461–29474 (2000).

[10] Gray, L. J. *et al.* Solar influences on climate. *Rev. Geophysics* **48**, RG4001 (2010).

[11] Haigh, J. D. The impact of solar variability on climate. *Science* **272**, 981–984 (1996).

[12] Ineson, S. *et al.* Solar forcing of winter climate variability in the Northern Hemisphere. *Nature Geosci.* **4**, 753–757 (2011).

[13] Lockwood, M. Solar influence on global and regional climates. *Surveys in Geophysics* **33**, 503–534 (2012).

[14] Fleitmann, D. *et al.* Holocene forcing of the Indian monsoon recorded in a stalagmite from southern Oman. *Science* **300**, 1737–1739 (2003).

[15] Adolphi, F. *et al.* Persistent link between solar activity and Greenland climate during the Last Glacial Maximum. *Nature Geosci.* **7**, 662–666 (2014).

[16] Wirth, S. B., Glur, L., Gilli, A. & Anselmetti, F. S. Holocene flood frequency across the Central Alps–solar forcing and evidence for variations in North Atlantic atmospheric circulation. *Quatern. Sci. Rev.* **80**, 112–128 (2013).

[17] Rimbu, N., Lohmann, G., Ionita, M., Czymzik, M. & Brauer, A. Interannual to millennial-scale variability of River Ammer floods and its relationship with solar forcing. *Int. J. Climatology* **41**, E644–E655 (2021).

[18] Moreno, A., Valero-Garcés, B. L., González-Sampériz, P. & Rico, M. Flood response to rainfall variability during the last 2000 years inferred from the Taravilla Lake record (Central Iberian Range, Spain). *J. Paleolimnol.* **40**, 943–961 (2008).

[19] Peña, J. C., Schulte, L., Badoux, A., Barriendos, M. & Barrera-Escoda, A. Influence of solar forcing, climate variability and modes of low-frequency atmospheric variability on summer floods in Switzerland. *Hydrol. Earth System Sci.* **19**, 3807–3827 (2015).

[20] Rimbu, N., Czymzik, M., Ionita, M., Lohmann, G. & Brauer, A. Atmospheric circulation patterns associated with the variability of River Ammer floods: evidence from observed and proxy data. *Clim. Past* **12**, 377–385 (2016).

[21] Saarni, S., Muschitiello, F., Weege, S., Brauer, A. & Saarinen, T. A late Holocene record of solar-forced atmospheric blocking variability over Northern Europe inferred from varved lake sediments of Lake Kuninkaisenlampi. *Quatern. Sci. Rev.* **154**, 100–110 (2016).

[22] Moffa-Sánchez, P., Born, A., Hall, I. R., Thornalley, D. J. & Barker, S. Solar forcing of North Atlantic surface temperature and salinity over the past millennium. *Nature Geosci.* **7**, 275–278 (2014).

[23] Kautz, L.-A. *et al.* Atmospheric blocking and weather extremes over the Euro-Atlantic sector–a review. *Weather Clim. Dyn.* **3**, 305–336 (2022).

[24] Wiedenmann, J. M., Lupo, A. R., Mokhov, I. I. & Tikhonova, E. A. The climatology of blocking anticyclones for the Northern and Southern Hemispheres: Block intensity as a diagnostic. *J. Clim.*

**15**, 3459–3473 (2002).

[25] Wilcox, P. S., Mudelsee, M., Spötl, C. & Edwards, R. L. Solar forcing of ENSO on century timescales. *Geophys. Res. Let.* **50**, e2023GL105201 (2023).

[26] Brönnimann, S., Xoplaki, E., Casty, C., Pauling, A. & Luterbacher, J. ENSO influence on Europe during the last centuries. *Clim. Dyn.* **28**, 181–197 (2007).

[27] Crabtree, P. J. & Bogucki, P. I. *Ancient Europe 8000 BC–1000 AD: An Encyclopedia of the Barbarian World* (Charles Scribner's Sons, 2004).

[28] Fowler, C., Harding, J. & Hofmann, D. *The Oxford Handbook of Neolithic Europe* (Oxford University Press, Oxford, 2015).

[29] Haak, W. *et al.* Massive migration from the steppe was a source for Indo-European languages in Europe. *Nature* **522**, 207 (2015).

[30] Allentoft, M. E. *et al.* Population genomics of post-glacial western Eurasia. *Nature* **625**, 301–311 (2024).

[31] Özdoğan, M. Archaeological evidence on the westward expansion of farming communities from eastern Anatolia to the Aegean and the Balkans. *Curr. Anthropol.* **52**, S415–S430 (2011).

[32] Horejs, B. *et al.* The Aegean in the early 7th millennium BC: maritime networks and colonization. *J. World Prehistory* **28**, 289–330 (2015).

[33] Lemmen, C. & Gronenborn, D. in *The diffusion of humans and cultures in the course of the spread of farming* (eds Bunde, A., Caro, J., Kärger, J. & Vogl, G.) *Diffusive Spreading in Nature, Technology and Society* 333–349 (Springer, 2018).

[34] Gronenborn, D. *et al.* in *Inherent collapse? social dynamics and external forcing in early neolithic and modern sw germany* (eds Riede, F. & Sheets, P. D.) *Going Forward by Looking Back: Archaeological Perspectives on Socio-Ecological Crisis, Response, and Collapse* 333–366 (Berghahn, New York, Oxford, 2020).

[35] Whittle, A., Healy, F. & Bayliss, A. *Gathering time: dating the early Neolithic enclosures of southern Britain and Ireland* (Oxbow Books, 2011).

[36] Andersson, M., Artursson, M. & Brink, K. The Funnel Beaker Culture in action: Early and Middle Neolithic monumentality in Southwestern Scania, Sweden (4000–3000 calBC). *J.Neolithic Archaeol.*

**24**, 61–97 (2022).

[37] Bunbury, M. M. E. *et al.* Understanding climate resilience in Scandinavia during the Neolithic and Early Bronze Age. *Quatern. Sci. Rev.* **322**, 108391 (2023).

[38] Klassen, L. *Jade und Kupfer: Untersuchungen zum Neolithisierungsprozess im westlichen Ostseeraum unter besonderer Berücksichtigung der Kulturentwicklung Europas 5500-3500 BC* Vol. 47 (Aarhus Universitetsforlag, 2004).

[39] Pétrequin, P. *et al.* *JADE: grandes haches alpines du Néolithique européen, Ve et IVe millénaires av. J.-C.* (Centre de Recherche Archéologique de la Vallée de l'Ain, 2012).

[40] Mittnik, A. *et al.* The genetic prehistory of the Baltic Sea region. *Nature Comm.* **9**, 442 (2018).

[41] Guilaine, J. La diffusion de l'agriculture en Europe : une hypothèse arythmique. *Zephyrus* **53-54**, 267–272 (2001).

[42] Lemmen, C. & Wirtz, K. W. On the sensitivity of the simulated European Neolithic transition to climate extremes. *J. Archaeol. Sci.* **51**, 65–72 (2014).

[43] Gronenborn, D. in *Climate Fluctuations, Human Migrations, and the Spread of Farming in Western Eurasia* (eds Biehl, P. F. & Nieuwenhuyse, O. P.) *Climate and cultural change in prehistoric Europe and the Near East* 211–232 (State University of New York, Albany, 2016).

[44] Silva, F. & Vander Linden, M. Amplitude of travelling front as inferred from 14C predicts levels of genetic admixture among European early farmers. *Sci. Rep.* **7**, 11985 (2017).

[45] Hinz, M., Feeser, I., Sjögren, K.-G. & Müller, J. Demography and the intensity of cultural activities: an evaluation of funnel beaker societies (4200–2800 cal bc). *J. Archaeol. Sci.* **39**, 3331–3340 (2012).

[46] Sánchez Goñi, M. F. *et al.* The expansion of Central and Northern European Neolithic populations was associated with a multi-century warm winter and wetter climate. *Holocene* **26**, 1188–1199 (2016).

[47] Martínez-Grau, H., Morell-Rovira, B. & Antolín, F. Radiocarbon dates associated to Neolithic contexts (Ca. 5900-2000 cal BC) from the northwestern Mediterranean arch to the high Rhine area. *J. Open Archaeol. Data* **9**, 1–10 (2021).

[48] Palmisano, A., Bevan, A., Kabelindde, A., Roberts, N. & Shennan, S. AIDA (Archive of Italian radiocarbon DAtes) **DOI:10.5281/zenodo.5846835** (2022).

[49] Perrin, T. Bda: une base de données archéologique collaborative en ligne. *Bul. Soc. préhist. française* **116**, 159–162 (2019).

[50] Schmid, C., Seidensticker, D. & Hinz, M. c14bazAAR: An R package for downloading and preparing C14 dates from different source databases. *J Open Source Soft.* **4**, 1914 (2019).

[51] Weninger, B., Jöris, O. & Danzeglocke, U. Cologne Radiocarbon Calibration & Palaeoclimate Research Package (2011). URL http://www.calpal.de.

[52] Capuzzo, G., Boaretto, E. & Barceló, J. A. EUBAR: A database of 14C measurements for the European Bronze Age. A Bayesian analysis of 14C-dated archaeological contexts from Northern Italy and Southern France. *Radiocarbon* **56**, 851–869 (2014).

[53] Shennan, S. *et al.* Regional population collapse followed initial agriculture booms in mid-Holocene Europe. *Nat. Commun* **4**, 2486 (2013).

[54] Manning, K., Colledge, S., Crema, E., Shennan, S. & Timpson, A. The cultural evolution of Neolithic Europe. EUROEVOL dataset 1: Sites, phases and radiocarbon data. *J Open Archaeol. Data* **5** (2016).

[55] Hinz, M. *et al.* RADON-Radiocarbon dates online 2012. Central European database of 14C dates for the Neolithic and the Early Bronze Age. *J. Neolithic Archaeol.* 10.12766/jna.2012.65 (2012).

[56] Chapple, R. M. Catalogue of radiocarbon determinations & dendrochronology dates (2018).

[57] Katsianis, M., Bevan, A., Styliaras, G. & Maniatis, Y. An Aegean history and archaeology written through radiocarbon dates. *J. Open Archaeol. Data* **8** (2020).

[58] Lucarini, G. *et al.* The MedAfriCarbon radiocarbon database and web application. Archaeological dynamics in Mediterranean Africa, ca. 9600-700 BC **DOI:10.5281/zenodo.3689716** (2020).

[59] Kondor, D. *et al.* Explaining population booms and busts in Mid-Holocene Europe. *Sci. Rep.* **13**, 9310 (2023).

[60] Palmisano, A., Bevan, A., Lawrence, D. & Shennan, S. NERD (Near East Radiocarbon Dates) **DOI:10.5281/zenodo.5767862** (2021).

[61] d'Errico, F., Banks, W. E., Vanhaeren, M., Laroulandie, V. & Langlais, M. PACEA geo-referenced radiocarbon database. *PaleoAnthropology* **2011**, 1–12 (2011).

[62] Palmisano, A., Bevan, A. & Shennan, S. Regional demographic trends and settlement patterns in central Italy: archaeological sites and radiocarbon dates. *J.Open Archaeol. Data* **6** (2018).

[63] Bird, D. *et al.* p3k14c, a synthetic global database of archaeological radiocarbon dates. *Scientific Data* **9**, 27 (2022).

[64] Rinne, C. *et al.* Radon-B. https://radonb.ufg.uni-kiel.de (2024).

[65] Bond, G. *et al.* Persistent solar influence on North Atlantic climate during the Holocene. *Science* **294**, 2130–2136 (2001).

[66] McDermott, F. Palaeo-climate reconstruction from stable isotope variations in speleothems: a review. *Quatern. Sci. Rev.* **23**, 901–918 (2004).

[67] Leuschner, H. H., Sass-Klaassen, U., Jansma, E., Baillie, M. G. & Spurk, M. Subfossil European bog oaks: population dynamics and long-term growth depressions as indicators of changes in the Holocene hydro-regime and climate. *Holocene* **12**, 695–706 (2002).

[68] Wassenburg, J. A. *et al.* Reorganization of the North Atlantic Oscillation during early Holocene deglaciation. *Nature Geosci.* **9**, 602 (2016).

[69] Macklin, M. G., Bonsall, C., Davies, F. M. & Robinson, M. R. Human–environment interactions during the Holocene: new data and interpretations from the Oban area, Argyll, Scotland. *Holocene* **10**, 109–121 (2000).

[70] Ait Brahim, Y. *et al.* North Atlantic ice-rafting, ocean and atmospheric circulation during the Holocene: Insights from Western Mediterranean speleothems. *Geophys. Res. Let.* **46**, 7614–7623 (2019).

[71] Baldini, L. M. *et al.* Regional temperature, atmospheric circulation, and sea-ice variability within the Younger Dryas Event constrained using a speleothem from northern Iberia. *Earth and Planet. Sci. Let.* **419**, 101–110 (2015).

[72] Ramos-Román, M. J. *et al.* Millennial-scale cyclical environment and climate variability during the Holocene in the western Mediterranean region deduced from a new multi-proxy analysis from the Padul record (Sierra Nevada, Spain). *Global Planet. Change* **168**, 35–53 (2018).

[73] Smith, A. C. *et al.* North Atlantic forcing of moisture delivery to Europe throughout the Holocene. *Sci. Rep.* **6**, 24745 (2016).

[74] Domínguez-Villar, D., Wang, X., Krklec, K., Cheng, H. & Edwards, R. L. The control of the tropical North Atlantic on Holocene millennial climate oscillations. *Geology* **45**, 303–306 (2017).

[75] Langdon, P., Barber, K. & Hughes, P. A 7500-year peat-based palaeoclimatic reconstruction and evidence for an 1100-year cyclicity in bog surface wetness from Temple Hill Moss, Pentland Hills, southeast Scotland. *Quaternary Science Reviews* **22**, 259–274 (2003).

[76] Macklin, M. G. *et al.* Past hydrological events reflected in the Holocene fluvial record of Europe. *Catena* **66**, 145–154 (2006).

[77] Atkinson, T. & Hopley, P. J. in *Speleothems and palaeoclimates* (eds Waltham, T. & Lowe, D.) *Caves and Karst of the Yorkshire Dales* 181–186 (British Cave Research Association, Buxton, UK, 2013).

[78] Daley, T. J. *et al.* The 8200 yr BP cold event in stable isotope records from the North Atlantic region. *Glob. Planetary Change* **79**, 288–302 (2011).

[79] Mary, Y. *et al.* Changes in Holocene meridional circulation and poleward Atlantic flow: the Bay of Biscay as a nodal point. *Clim. Past* **13**, 201–216 (2017).

[80] Davis, B. A. S., Brewer, S., Stevenson, A. C. & Guiot, J. The temperature of Europe during the Holocene reconstructed from pollen data. *Quatern. Sci. Rev.* **22**, 1701–1716 (2003).

[81] Marsicek, J., Shuman, B. N., Bartlein, P. J., Shafer, S. L. & Brewer, S. Reconciling divergent trends and millennial variations in Holocene temperatures. *Nature* **554**, 92–96 (2018).

[82] Moreno, A. *et al.* New speleothem data from Molinos and Ejulve caves reveal Holocene hydrological variability in northeast Iberia. *Quatern. Res.* **88**, 223–233 (2017).

[83] Johnstone, E., Macklin, M. G. & Lewin, J. The development and application of a database of radiocarbon-dated Holocene fluvial deposits in Great Britain. *Catena* **66**, 14–23 (2006).

[84] Leunda, M., Gil-Romera, G., Daniau, A.-L., Benito, B. M. & González-Sampériz, P. Holocene fire and vegetation dynamics in the Central Pyrenees (Spain). *Catena* **188**, 104411 (2020).

[85] Wainer, K. *et al.* Speleothem record of the last 180 ka in Villars cave (SW France): Investigation of a large $\delta$18O shift between MIS6 and MIS5. *Quatern. Sci. Rev.* **30**, 130–146 (2011).

[86] McDermott, F. *et al.* Holocene climate variability in Europe: evidence from $\delta$18O, textural and extension-rate variations in three speleothems. *Quatern. Sci. Rev.* **18**, 1021–1038 (1999).

[87] Frigola, J. *et al.* Holocene climate variability in the western Mediterranean region from a deepwater sediment record. *Paleoceanography* **22**, PA2209 (2007).

[88] Ruan, J. *et al.* Evidence of a prolonged drought ca. 4200 yr BP correlated with prehistoric settlement abandonment from the Gueldaman GLD1 Cave, Northern Algeria. *Clim. Past* **12**, 1–14 (2016).

[89] Genty, D. & Massault, M. Carbon transfer dynamics from bomb-14C and $\delta$13C time series of a laminated stalagmite from SW France—modelling and comparison with other stalagmite records. *Geochim. Cosmochim. Acta* **63**, 1537–1548 (1999).

[90] Nesje, A., Matthews, J. A., Dahl, S. O., Berrisford, M. S. & Andersson, C. Holocene glacier fluctuations of Flatebreen and winter-precipitation changes in the Jostedalsbreen region, western Norvay, based on glaciolacustrine sediment records. *Holocene* **11**, 267–280 (2001).

[91] Litt, T., Schoelzel, C., Kuehl, N. & Brauer, A. Vegetation and climate history in the Westeifel Volcanic Field (Germany) during the past 11 000 years based on annually laminated lacustrine maar sediments. *Boreas* **38**, 679–690 (2009).

[92] Magny, M. Holocene climate variability as reflected by mid-European lake-level fluctuations and its probable impact on prehistoric human settlements. *Quatern. Int.* **113**, 65–79 (2004).

[93] Affolter, S. *et al.* Central Europe temperature constrained by speleothem fluid inclusion water isotopes over the past 14,000 years. *Sci. Adv.* **5**, eaav3809 (2019).

[94] Fohlmeister, J. *et al.* Bunker Cave stalagmites: an archive for central European Holocene climate variability. *Clim. Past* **8**, 1751–1764 (2012).

[95] Niggemann, S., Mangini, A., Mudelsee, M., Richter, D. K. & Wurth, G. Sub-Milankovitch climatic cycles in Holocene stalagmites from Sauerland, Germany. *Earth Planet. Sci. Let.* **216**, 539–547 (2003).

[96] Wick, L., van Leeuwen, J., van der Knaap, W. & Lotter, A. Holocene vegetation development in the catchment of Sägistalsee (1935 m asl), a small lake in the Swiss Alps. *J. Paleolimnol.* **30**, 261–272 (2003).

[97] Luetscher, M., Hoffmann, D., Frisia, S. & Spötl, C. Holocene glacier history from alpine speleothems, Milchbach cave, Switzerland. *Earth Planet. Sci. Let.* **302**, 95–106 (2011).

[98] Mischel, S. A. *et al.* Holocene climate variability in Central Germany and a potential link to the polar North Atlantic: A replicated record from three coeval speleothems. *Holocene* **27**, 509–525 (2017).

[99] Goslin, J. *et al.* Holocene centennial to millennial shifts in North-Atlantic storminess and ocean dynamics. *Sci. Rep.* **8**, 12778 (2018).

[100] Drysdale, R. *et al.* Late Holocene drought responsible for the collapse of Old World civilizations is recorded in an Italian cave flowstone. *Geology* **34**, 101–104 (2006).

[101] Regattieri, E. *et al.* Lateglacial to holocene trace element record (Ba, Mg, Sr) from corchia cave (Apuan Alps, central Italy): paleoenvironmental implications. *J. Quat. Sci.* **29**, 381–392 (2014).

[102] Isola, I. *et al.* Speleothem U/Th age constraints for the Last Glacial conditions in the Apuan Alps, northwestern Italy. *Paleogeogr. Palaeoclimatol. Palaeoecol.* **518**, 62–71 (2019).

[103] Baroni, C., Zanchetta, G., Fallick, A. E. & Longinelli, A. Mollusca stable isotope record of a core from Lake Frassino, northern Italy: hydrological and climatic changes during the last 14 ka. *Holocene* **16**, 827–837 (2006).

[104] Ilyashuk, E. A., Koinig, K. A., Heiri, O., Ilyashuk, B. P. & Psenner, R. Holocene temperature variations at a high-altitude site in the Eastern Alps: a chironomid record from Schwarzsee ob Sölden, Austria. *Quatern. Sci. Rev.* **30**, 176–191 (2011).

[105] von Grafenstein, U., Erlenkeuser, H., Müller, J., Jouzel, J. & Johnsen, S. The cold event 8200 years ago documented in oxygen isotope records of precipitation in Europe and Greenland. *Clim. Dyn.* **14**, 73–81 (1998).

[106] Scholz, D. *et al.* Holocene climate variability in north-eastern Italy: potential influence of the NAO and solar activity recorded by speleothem data. *Clim. Past* **8**, 1367–1383 (2012).

[107] Fohlmeister, J., Vollweiler, N., Spötl, C. & Mangini, A. COMNISPA II: Update of a mid-European isotope climate record, 11 ka to present. *Holocene* **23**, 749–754 (2013).

[108] Vollweiler, N., Scholz, D., Mühlinghaus, C., Mangini, A. & Spötl, C. A precisely dated climate record for the last 9 kyr from three high alpine stalagmites, Spannagel Cave, Austria. *Geophys. Res. Let.* **33**, L20703 (2006).

[109] Combourieu-Nebout, N. *et al.* Holocene vegetation and climate changes in the central Mediterranean inferred from a high-resolution marine pollen record (Adriatic Sea). *Clim. Past* **9**, 2023–2042 (2013).

[110] Borgmark, A. Holocene climate variability and periodicities in south-central Sweden, as interpreted from peat humification analysis. *Holocene* **15**, 387–395 (2005).

[111] Calò, C. *et al.* Spatio-temporal patterns of Holocene environmental change in southern Sicily. *Paleogeogr. Palaeoclimatol. Palaeoecol.* **323**, 110–122 (2012).

[112] Seppä, H., Hammarlund, D. & Antonsson, K. Low-frequency and high-frequency changes in temperature and effective humidity during the Holocene in south-central Sweden: implications for atmospheric and oceanic forcings of climate. *Climate Dynamics* **25**, 285–297 (2005).

[113] Seppä, H., Bjune, A. E., Telford, R. J., Birks, H. J. B. & Veski, S. Last nine-thousand years of temperature variability in Northern Europe. *Clim. Past* **5**, 523–535 (2009).

[114] Linge, H. *et al.* Stable isotope records for the last 10000 years from Okshola cave (Fauske, northern Norway) and regional comparisons. *Clim. Past* **5**, 667–682 (2009).

[115] Peyron, O. *et al.* Contrasting patterns of climatic changes during the Holocene in the Central Mediterranean (Italy) reconstructed from pollen data. *Clim. Past* **9**, 1233 – 1252 (2013).

[116] Rubensdotter, L. & Rosqvist, G. The effect of geomorphological setting on Holocene lake sediment variability, northern Swedish Lapland. *J. Quat. Sci.* **18**, 757–767 (2003).

[117] Demény, A. *et al.* Mid-Holocene climate conditions and moisture source variations based on stable H, C and O isotope compositions of speleothems in Hungary. *Quatern. Int.* **293**, 150–156 (2013).

[118] Warden, L. *et al.* Climate induced human demographic and cultural change in northern Europe during the mid-Holocene. *Sci. Rep.* **7**, 15251 (2017).

[119] Frogley, M. R., Griffiths, H. I. & Heaton, T. H. Historical biogeography and Late Quaternary environmental change of Lake Pamvotis, Ioannina (north-western Greece): evidence from ostracods. *J. Biogeogr.* **28**, 745–756 (2001).

[120] Vogel, H., Wagner, B., Zanchetta, G., Sulpizio, R. & Rosén, P. A paleoclimate record with tephrochronological age control for the last glacial-interglacial cycle from Lake Ohrid, Albania and Macedonia. *J. Paleolimnol.* **44**, 295–310 (2010).

[121] Constantin, S., Bojar, A.-V., Lauritzen, S.-E. & Lundberg, J. Holocene and Late Pleistocene climate in the sub-Mediterranean continental environment: a speleothem record from Poleva Cave (Southern Carpathians, Romania). *Paleogeogr. Palaeoclimatol. Palaeoecol.* **243**, 322–338 (2007).

[122] Persoiu, A. *et al.* Holocene winter climate variability in Central and Eastern Europe. *Sci. Rep.* **7**, 1196 (2017).

[123] Drăguşin, V. *et al.* Constraining Holocene hydrological changes in the Carpathian–Balkan region using speleothem $\delta^{18}$O and pollen-based temperature reconstructions. *Clim. Past* **10**, 1363–1380 (2014).

[124] Dobrowolski, R. *et al.* Multi-proxy evidence of Holocene climate variability in Volhynia Upland (SE Poland) recorded in spring-fed fen deposits from the Komarów site. *Holocene* **26**, 1406–1425 (2016).

[125] Psomiadis, D., Dotsika, E., Albanakis, K., Ghaleb, B. & Hillaire-Marcel, C. Speleothem record of climatic changes in the northern Aegean region (Greece) from the Bronze Age to the collapse of the Roman Empire. *Paleogeogr. Palaeoclimatol. Palaeoecol.* **489**, 272–283 (2018).

[126] Giesecke, T. *et al.* Exploring Holocene continentality changes in Fennoscandia using present and past tree distributions. *Quatern. Sci. Rev.* **27**, 1296–1308 (2008).

[127] Husum, K. & Hald, M. A continuous marine record 8000-1600 cal. yr BP from the Malangenfjord, north Norway: foraminiferal and isotopic evidence. *Holocene* **14**, 877–887 (2004).

[128] Stansell, N. D. *et al.* A stable isotope record of Holocene precipitation dynamics in the Baltic region from Lake Nuudsaku, Estonia. *Quatern. Sci. Rev.* **175**, 73–84 (2017).

[129] Heikkilä, M. & Seppä, H. A 11,000yr palaeotemperature reconstruction from the southern boreal zone in Finland. *Quatern. Sci. Rev.* **22**, 541–554 (2003).

[130] Seppä, H. & Poska, A. Holocene annual mean temperature changes in Estonia and their relationship to solar insolation and atmospheric circulation patterns. *Quatern. Res.* **61**, 22–31 (2004).

[131] Sperling, M. *et al.* Black Sea impact on the formation of eastern Mediterranean sapropel S1? Evidence from the Marmara Sea. *Paleogeogr. Palaeoclimatol. Palaeoecol.* **190**, 9–21 (2003).

[132] Fleitmann, D. *et al.* Timing and climatic impact of Greenland interstadials recorded in stalagmites from northern Turkey. *Geophys. Res. Let.* **36** (2009).

[133] Woodbridge, J. *et al.* Pollen-inferred regional vegetation patterns and demographic change in Southern Anatolia through the Holocene. *Holocene* **29**, 728–741 (2019).

[134] Jones, V. J., Leng, M. J., Solovieva, N., Sloane, H. J. & Tarasov, P. Holocene climate of the Kola Peninsula; evidence from the oxygen isotope record of diatom silica. *Quatern. Sci. Rev.* **23**, 833–839 (2004).

[135] Roberts, N. *et al.* Stable isotope records of Late Quaternary climate and hydrology from Mediterranean lakes: the ISOMED synthesis. *Quatern. Sci. Rev.* **27**, 2426–2441 (2008).

[136] Bar-Matthews, M., Ayalon, A., Kaufman, A. & Wasserburg, G. J. The Eastern Mediterranean paleoclimate as a reflection of regional events: Soreq cave, Israel. *Earth Planet. Sci. Let.* **166**, 85–95 (1999).

[137] Hinz, M., Schirrmacher, J., Kneisel, J., Rinne, C. & Weinelt, M. The Chalcolithic–Bronze Age transition in southern Iberia under the influence of the 4.2 kyr event? A correlation of climatological and demographic proxies. *J. Neolithic Archaeol.* **21**, 1–26 (2019).

[138] Risch, R., Friederich, S., Küssner, M. & Meller, H. Architecture and settlement dynamics in central germany from the late neolithic to the early bronze age. *Proc. Prehist. Soc.* **88**, 123–154 (2022).

[139] Bunting, M. J., Farrell, M., Bayliss, A., Marshall, P. & Whittle, A. Maps from mud—using the multiple scenario approach to reconstruct land cover dynamics from pollen records: a case study of two Neolithic landscapes. *Frontiers Ecol. Evol.* **6**, 36 (2018).

[140] Berger, J.-F. *et al.* Holocene land cover and population dynamics in Southern France. *Holocene* **29**, 776–798 (2019).

[141] Kleijne, J., Weinelt, M. & Müller, J. Late Neolithic and Chalcolithic maritime resilience? The 4.2 ka BP event and its implications for environments and societies in Northwest Europe. *Env. Res. Let.* **15**, 125003 (2020).

[142] Zimmermann, A., Hilpert, J. & Wendt, K. P. Estimations of population density for selected periods between the Neolithic and AD 1800. *Human Biology* **81**, 357–380 (2009).

[143] Heitz, C., Laabs, J., Hinz, M. & Hafner, A. in *Collapse and resilience in prehistoric archaeology: Questioning concepts and causalities in models of climate-induced societal transformations* (eds Erdkamp, P., Manning, J. G. & Verboven, K.) *Climate Change and Ancient Societies in Europe and the Near East: Diversity in Collapse and Resilience* 127–199 (Springer, 2021).

[144] Gronenborn, D., Strien, H.-C. & Lemmen, C. Population dynamics, social resilience strategies, and Adaptive Cycles in early farming societies of SW Central Europe. *Quatern. Int.* **446**, 54–65 (2017).

[145] Peters, R. & Zimmermann, A. Resilience and cyclicity: towards a macrohistory of the Central European Neolithic. *Quatern. Int.* **446**, 43–53 (2017).

[146] Gronenborn, D., Strien, H.-C., Dietrich, S. & Sirocko, F. 'Adaptive cycles' and climate fluctuations: a case study from Linear Pottery Culture in western Central Europe. *J. Archaeol. Sci.* **51**, 73–83 (2014).

[147] Palmisano, A., Bevan, A. & Shennan, S. Comparing archaeological proxies for long-term population patterns: An example from central Italy. *J. Archaeol. Sci.* **87**, 59–72 (2017).

[148] Feeser, I., Dörfler, W., Kneisel, J., Hinz, M. & Dreibrodt, S. Human impact and population dynamics in the Neolithic and Bronze Age: Multi-proxy evidence from north-western Central Europe. *Holocene* **29**, 1596–1606 (2019).

[149] Tkáč, P. & Kolář, J. Towards New Demography Proxies and Regional Chronologies: Radiocarbon Dates from Archaeological Contexts Located in the Czech Republic Covering the Period Between 10,000 BC and AD 1250. *J. Open Archaeol. Data* **9**, DOI:10.5334/joad.85 (2021).

[150] Weiberg, E. *et al.* Long-term trends of land use and demography in Greece: A comparative study. *Holocene* **29**, 742–760 (2019).

[151] Harper, T. K. *et al.* Combining Relative Chronology and AMS 14C Dating to Contextualize 'Megasites', Serial Migrations and Diachronic Expressions of Material Culture in the Western Tripolye Culture, Ukraine. *Docum. Praehistor.* **48**, 276–296 (2021).

[152] Brigand, R. & Weller, O. Kernel density estimation and transition maps of Moldavian Neolithic and Eneolithic settlement. *Data in Brief* **17**, 452–458 (2018).

[153] Blum, S. W., Thater, M. & Thumm-Doğrayan, D. Pernicka, E., Rose, C. & Jablonka, P. (eds) *Die Besiedlung der Troas vom Neolithikum bis zum Beginn der mittleren Bronzezeit: Chronologische Sequenz und Siedlungsstruktur.* (eds Pernicka, E., Rose, C. & Jablonka, P.) *Studia Troica, Monographien 5. Troia 1987-2012: Grabungen und Forschungen I*, 770–863 (Universität Tübingen, 2014).

[154] Diachenko, A. in *Demography reloaded* (eds Müller, J., Rassmann, K. & Videiko, M.) *Trypillia mega-sites and European prehistory: 4100-3400 BCE* 181–193 (Routledge, 2016).

[155] Ohlrau, R. Trypillia Mega-Sites: Neither Urban nor Low-Density? *J. Urban Archaeol.* **5**, 81–100 (2022).

[156] Meller, H., Risch, R., Jung, R. & Arz, R. (eds). *The 4.2 ka BP climatic event in west and central Anatolia: combining palaeo-climatic proxies and archaeological data* (2015).
